# Regulatory architecture of housekeeping genes is driven by promoter assemblies

**DOI:** 10.1016/j.celrep.2023.112505

**Published:** 2023-05-12

**Authors:** Marion Dejosez, Alessandra Dall’Agnese, Mahesh Ramamoorthy, Jesse Platt, Xing Yin, Megan Hogan, Ran Brosh, Abraham S. Weintraub, Denes Hnisz, Brian J. Abraham, Richard A. Young, Thomas P. Zwaka

**Affiliations:** 1 Black Family Stem Cell Institute, Huffington Center for Cell-based Research in Parkinson’s Disease, Department of Cell, Developmental and Regenerative Biology, Icahn School of Medicine at Mount Sinai, New York, NY 10502, USA; 2 Whitehead Institute for Biomedical Research, Cambridge, MA 02142, USA; 3 Department of Biology, Massachusetts Institute of Technology, Cambridge, MA 02142, USA; 4 St. Jude Research Children’s Hospital, Memphis, TN 38105, USA; 5 Senior author; 6 These authors contributed equally; 7 Lead contact

## Abstract

Genes that are key to cell identity are generally regulated by cell-type-specific enhancer elements bound by transcription factors, some of which facilitate looping to distant gene promoters. In contrast, genes that encode housekeeping functions, whose regulation is essential for normal cell metabolism and growth, generally lack interactions with distal enhancers. We find that Ronin (Thap11) assembles multiple promoters of housekeeping and metabolic genes to regulate gene expression. This behavior is analogous to how enhancers are brought together with promoters to regulate cell identity genes. Thus, Ronin-dependent promoter assemblies provide a mechanism to explain why housekeeping genes can forgo distal enhancer elements and why Ronin is important for cellular metabolism and growth control. We propose that clustering of regulatory elements is a mechanism common to cell identity and housekeeping genes but is accomplished by different factors binding distinct control elements to establish enhancer-promoter or promoter-promoter interactions, respectively.

## In brief

Using mouse embryonic stem cells, Dejosez et al. describe how Ronin enables promoter-promoter interactions to regulate housekeeping gene expression, bypassing distal enhancers. This mechanism is analogous to promoter-enhancer interactions that regulate cell identity genes and highlights Ronin’s importance in cellular metabolism and growth.

## Graphical Abstract

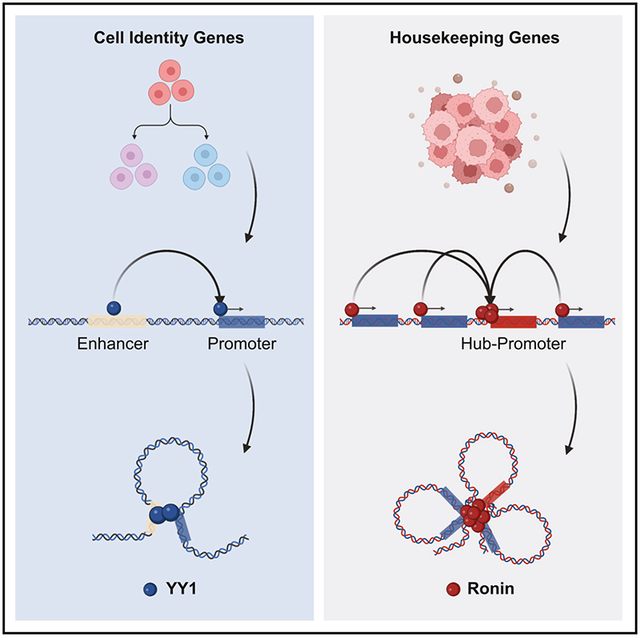

## INTRODUCTION

Gene regulation and chromosome architecture are intimately linked in eukaryotes.^1-4^ In the case of cell-type-specific genes, transcription factors bind to enhancer elements and super-enhancers and act to regulate transcription of distal target genes by coming into physical proximity through three-dimensional (3D) looping of the intervening DNA.^5-12^ Some transcription factors that bind these elements and are capable of multimerization, such as YY1 in mammalian cells and GAGA in *Drosophila*, can act as DNA looping factors to facilitate this process.^13-17^ Furthermore, cell-type-specific genes are often engaged by more than one enhancer element,^18-25^ and this clustering of enhancers and promoters is associated with biophysical processes that concentrate transcription factors and coactivators into transcriptional condensates.^26-40^ However, this fundamental process, in which sequence information embedded in DNA forms the basis for assembling nucleoproteins that regulate cell-type-specific genes, has not been recognized to occur in genes that are less frequently associated with enhancers, such as the housekeeping genes that are essential for metabolism and maintenance of other fundamental processes in all cells.

Of the approximately 20,000 protein-coding genes encoded in mammalian genomes, approximately 9,000 have been annotated as housekeeping genes (Human Protein Atlas^41^). Housekeeping genes sometimes have promoter-proximal enhancers, but their promoters frequently lack interactions with distal enhancers, suggesting that the mechanisms involved in enhancer-promoter DNA loops may not generally contribute to housekeeping gene regulation.^23,37,42,43^ It has long been thought that the reason for the apparent absence of enhancer-promoter interactions is that housekeeping genes are generally expressed and thus do not require the regulatory finesse provided by interactions among enhancers and cell-type-specific genes. However, it is important to bear in mind that expression of many of these genes is finely tuned to respond to the changing metabolic demands of cells^44^ and that they are often co-regulated in groups within clusters.^45^ Therefore, one question that still needs to be answered is how transcription of housekeeping genes is controlled if not by enhancer-promoter-based DNA looping.

**Figure 1.**
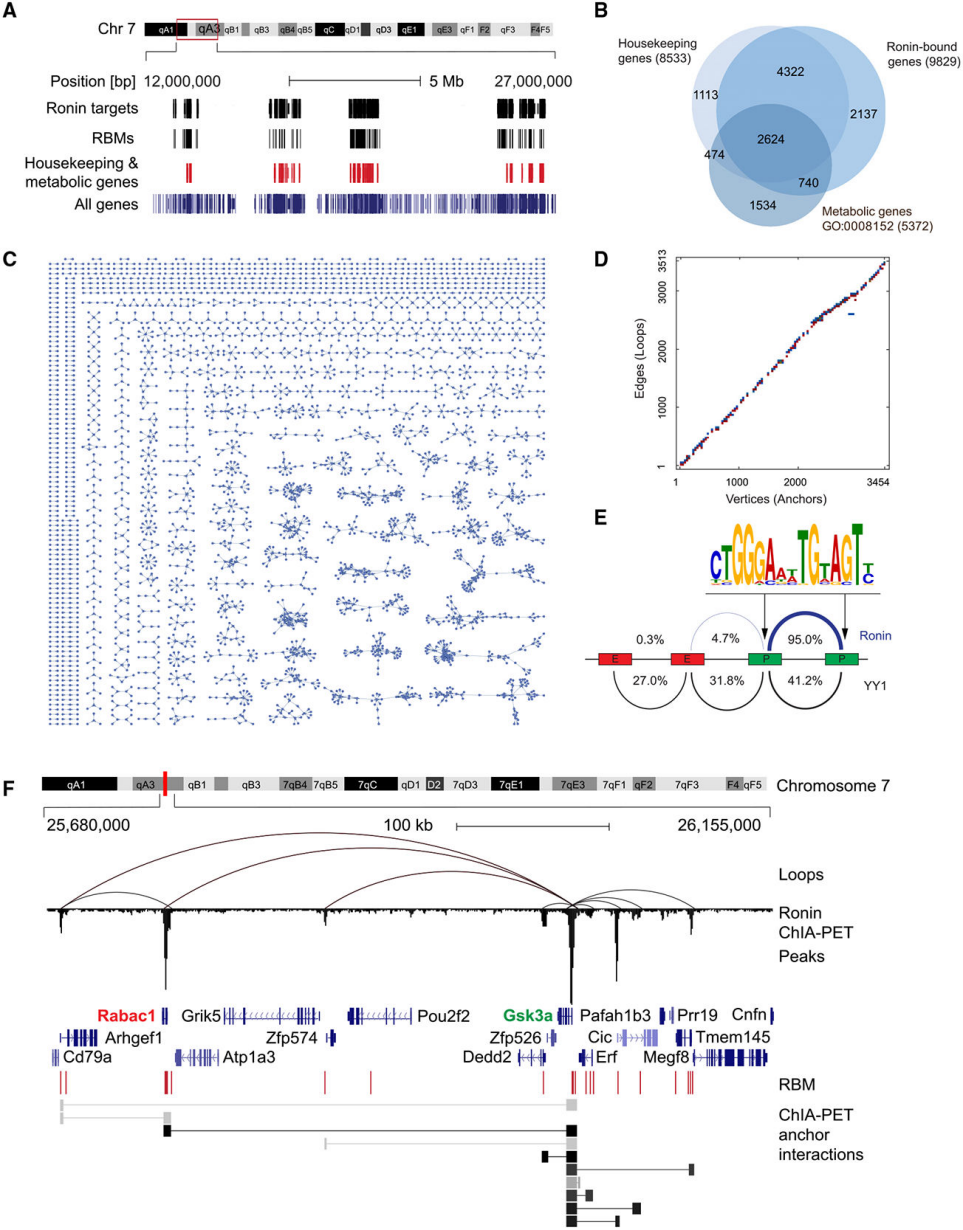
Ronin arranges core promoters into hierarchical clusters (A) Visualization of Ronin target genes, Ronin-binding motifs (RBMs), and their overlap with previously reported housekeeping and metabolic genes throughout a larger region on chromosome 7. All genes are shown as reference. (B) Venn diagram of protein-coding genes that are bound by Ronin, housekeeping genes, and metabolic genes. Housekeeping genes are the combined set of mouse homologs of the human housekeeping genes annotated by Eisenberg and Levanon^41^ and by the Human Protein Atlas. Metabolic genes are defined by Panther GO:0008152. Statistical enrichment for housekeeping genes (p = 0) and metabolic genes (p = 0) was determined by hypergeometric testing. (C) Network depiction of all intrachromosomal Ronin-mediated DNA interactions identified by ChIA-PET. Shown are the merged interactions from two ChIA-PET experiments. (D) Incident plot depicting the interaction between vertices (anchors) and edges (loops) in the Ronin network; vertices are incident to an edge when the edge is connected to the vertex. (E) Proportion of Ronin-mediated DNA interactions involving enhancer (E) and promoter (P) elements (88% of all interactions) compared with the loop-forming transcription factor YY1 (85% of all interactions). Also shown is the RBM, found in a motif search within the newly identified Ronin-binding peaks; e = 1.6 × 10e–39. (F) Illustration of interactions formed between anchors around the “hierarchical” *Gsk3a* P on chromosome 7. Shown are the merged interactions from two ChIA-PET experiments. Interactions with more than 10 PETs are displayed. The arc plot only includes interactions that start and end in this region. Related to Figures S1-S3 and Tables S1 and S2.

One of the most prominent features of housekeeping genes, and an attribute that was recognized in the earliest whole-genome sequence studies, is that these genes tend to be grouped together locally within the genome (Figure 1A).^46-48^ This local organization and lack of long-distance interactions with enhancers raises the possibility that individual housekeeping genes might achieve productive transcription through contact of their promoters with each other instead of distal enhancers. Indeed, such a concept has been recently proposed for genes that tend to be adjacent to one another in the genome,^49^ but a molecular explanation of how this may arise and how broadly it applies is missing.

A strong candidate for a promoter sequence that might contribute to the control of housekeeping genes is the ultra-conserved M4 sequence, one of the most conserved sequence motifs in the human genome, also known as the Ronin-binding motif^50-55^ (RBM; CTGGGARWTGTAGTY). What makes the RBM relevant in the current context is that, unlike any enhancer motif, it maintains an extreme positional preference for locations immediately upstream of transcription start sites (TSSs; −81 bp on average) and away from cell-type-specific distal enhancers.^54,55^ Critically, RBMs are primarily associated with housekeeping genes, such as metabolic genes, that tend to be expressed in all cells.^50,51,56^ Furthermore, Ronin (Thap11), a highly conserved DNA transposon-derived transcription factor that binds to this sequence motif,^57-60^ is essential for many primary functions of cells, including stem cell growth and metabolism, but not for cell-type-specific fate decisions.^50,61,62^ However, Ronin’s molecular modus operandi has not yet been established.^63^

In this study, we show that housekeeping genes are regulated through promoter-promoter contacts in analogy to regulation of cell-type-specific genes that are regulated through enhancer-enhancer and enhancer-promoter interactions. Further, the promoter-promoter contacts depend on Ronin, which specializes in binding to the core promoters of many housekeeping genes. By binding and connecting multiple promoters, Ronin establishes promoter clusters that form transcriptional condensates to ensure adequate transcriptional control of housekeeping genes. Thus, our findings demonstrate that clustering of gene regulatory elements, whether enhancers or promoters, is a general principle of gene regulation that is not limited to cell identity genes but extends to housekeeping genes that are essential for the basic activities of all cells.

## RESULTS

### Ronin is associated with promoter-promoter contacts among housekeeping genes

To explore the putative link between Ronin and housekeeping genes, we first used chromatin interaction analysis by paired-end tag sequencing (ChIA-PET)^64^ to produce a highly detailed map of Ronin-binding sites in mouse embryonic stem cells (mESCs) (Figure 1A; Tables S1A-S1C). mESCs provided an ideal model for our studies because Ronin’s function has been best characterized in this cell type;^50,61,62^ they are genetically uncompromised, as opposed to tumor cell lines, and represent an authentic developmental *in vitro* model of the early embryonic “inner cell mass.” We found that Ronin was associated with the promoters of 10,916 genes (Table S1C) that showed a high degree of overlap with the much smaller set of previously described Ronin target genes (Figure S1A)^21,50^ and, as expected, were enriched with RBMs (Figure S1B; Table S1D). Of the target genes, 9,355 were protein-coding genes bound by Ronin at their promoters, which were substantially enriched in housekeeping and metabolic genes and processes (Figures 1B and S1C; Tables S1E-S1G). These results establish that Ronin broadly occupies core promoters of housekeeping genes, many of which are engaged in metabolic functions.

The presence of Ronin at the promoters of clustered housekeeping genes led us to test whether these sites serve as anchors for DNA contacts among these promoters. We generated detailed contact maps of DNA loops related to Ronin using the ChIA-PET data. This analysis identified 3,513 Ronin-bound sites that served as anchors for a total of 3,454 intrachromosomal interactions (looping events) (Figures 1C and S2; Tables S1A and S1H). While we also detected interchromosomal interactions (Tables S1A and S1H), they have been reported to have a greater amount of noise than intrachromosomal interactions,^65^ and, hence, we did not include them in our subsequent analyses. We found that most of the intrachromosomal interaction events were of short distances between 10 and 50 kb (Figures 1D and S3A). As expected, given the proximity of Ronin and its binding motif to TSSs (Figure S3B),^50,54,55^ a vast majority (~95%) of these interactions involved promoter-to-promoter associations, whereas only a few (~5%) involved enhancers (Figure 1E; Table S1H). This result contrasted with that of other transcription factors, such as YY1^17^ (Figure 1E), Sox2,^66^ nuclear factor κB (NF-κB),^67^ and Erα,^64^ for which a larger fraction of interactions involves enhancers. As predicted, genes that were bound and looped by Ronin were highly enriched in housekeeping and metabolic genes and functions (Figures S3C and S3D; Tables S1E-S1G). Thus, we concluded that Ronin is predominantly associated with promoter-promoter loops among housekeeping and metabolic genes.

Cell-type-specific genes are often regulated by multiple enhancers that contact one another as well as the promoters of their target genes. Hence, we next sought to determine whether such multivalency also applied to our identified promoter-promoter interactions. Further analysis of our ChIA-PET data revealed that, rather than just bringing adjacent gene promoters together, Ronin-associated loops clustered multiple housekeeping gene promoters into single units. Oftentimes one promoter within a given cluster stood out by virtue of its interactions with an especially large number of looped promoters. An instance of such a promoter cluster, in which several promoters were tethered, is the *Gsk3a* locus (Figure 1F, loops with ≥10 PETs are illustrated); the *Gsk3a* core promoter has 11 RBM sequences (Figure S4A) and is linked with 15 neighboring promoters (Tables S1B and S1H), creating what amounts to a highly dense promoter cluster. The *Ronin* (*Thap11*) gene locus itself (Figures S4B and S4C) and the *Mmachc* gene locus (Figure S4D) provided additional examples. Notably, the number of RBMs within promoters positively correlated with the number of loops formed (Figure S4E). A comparison of our contact maps with a published promoter-capture high-throughput chromosome conformation capture (Hi-C) dataset in mESCs^68^ validated our findings. We found that approximately 40% of Ronin-mediated promoter interactions were also engaged in promoter-promoter contacts identified through promoter-capture Hi-C (Table S1I). This statistically significant overlap is notable considering that the datasets were generated with different experimental systems that come with their own technical and analytical biases and that the mESCs were derived from different strains of mice and cultured in different media. The Gsk3a locus provides an example of multi-promoter assemblies that showed high degrees of overlap in these independent datasets (Figure S4F). In summary, these results demonstrate that Ronin is associated with promoter-promoter contacts among housekeeping genes.

**Figure 2.**
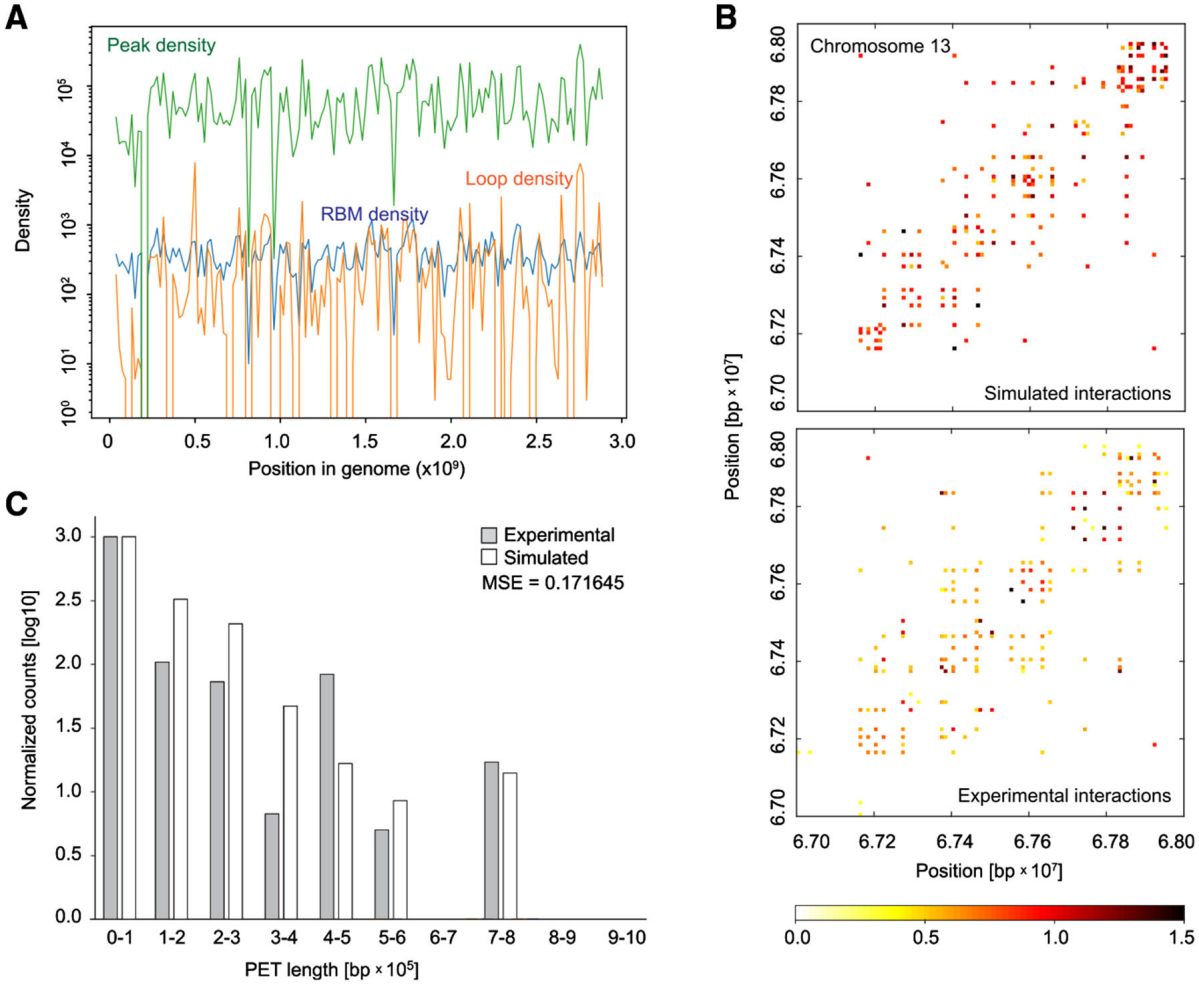
Prediction of Ronin-mediated loop formation based on RBM distribution (A) Correlation plots showing the relation between RBMs and peak and loop densities. The correlation coefficients between the RBMs and peak density and the RBMs and loop density are 0.801 and 0.353, respectively. (B) Heatmap of Ronin-associated interactions, simulated based on the distribution of RBMs in a representative region on chromosome 13 (“dephasing” distance [LD] = 10 nodes, simulation temperature [T] = 4,000 K, and time step [dt] = 0.15625 s; see STAR Methods for details) (top) compared with interactions experimentally detected by ChIA-PET in the same region (bottom). (C) Comparison of PET pair length distributions between the simulated (top) and experimental results (bottom). MSE, mean-squared error; PET, paired-end tag.

We next used computational modeling approaches to further elaborate our hypothesis that promoter looping is linked to the presence of RBMs. We first performed time-series analyses to determine the correlation between Ronin-binding and Ronin-mediated loop formation based on RBM distribution and found that they were correlated (Figure 2A). To corroborate the importance of the RBM in looping, we then performed coarse-grained molecular dynamics simulations of all chromosomes. For each pair of nodes, a force field was constructed, where we calculated the linear base pair distance between the two nodes. We then dephased the distance to characterize the “stiffness” of the chromosome and simulated 10,000 steps after an equilibrium was reached (see STAR Methods for details). This computational model was able to accurately predict the observed looping pattern solely based on the positions of Ronin-bound sequences (Figures 2B and 2C). These findings would be difficult to explain without invoking the idea that Ronin directly organizes the DNA looping events.

### Promoter-promoter interactions among housekeeping genes depend on Ronin

**Figure 3.**
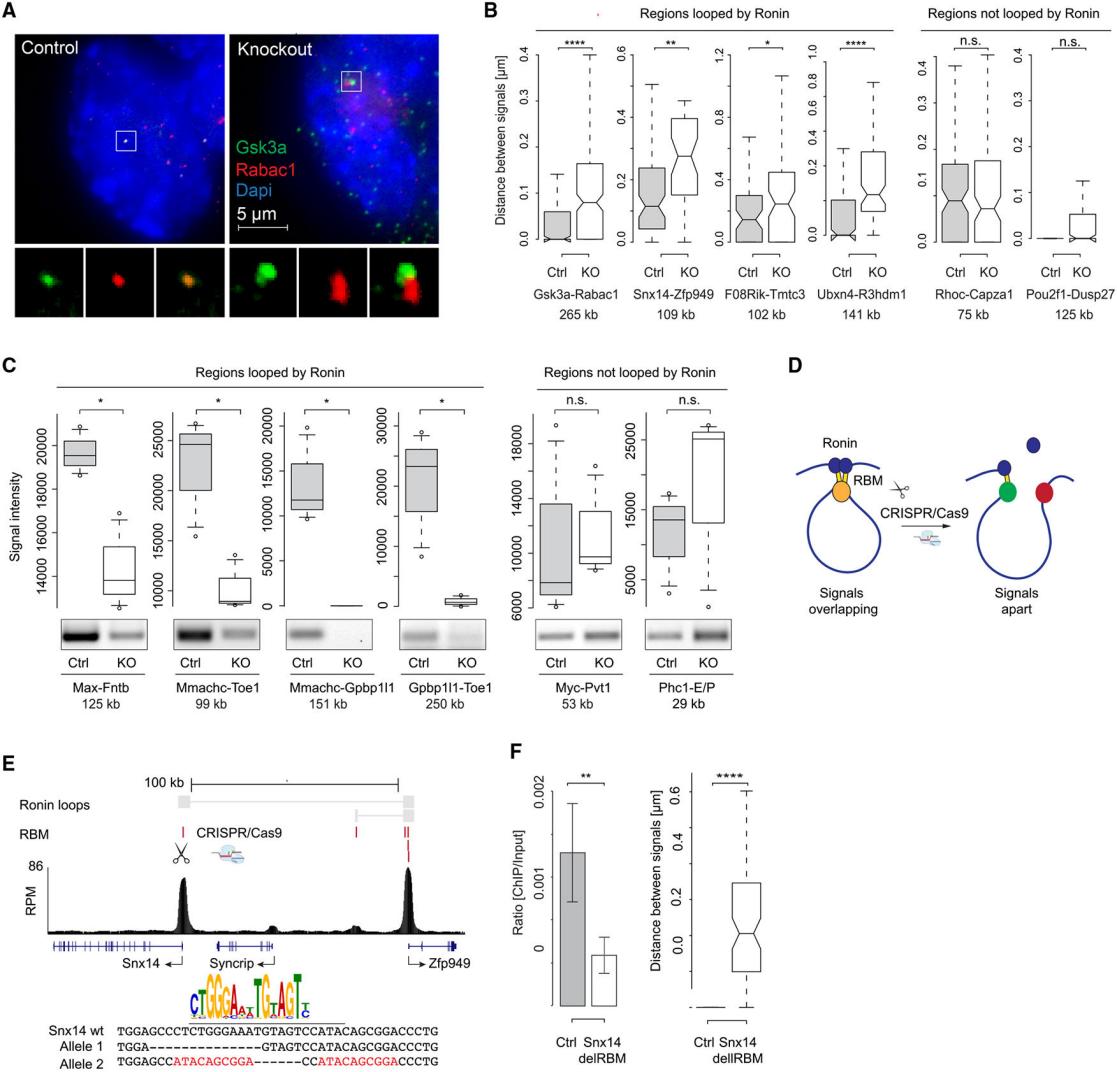
Ronin and the Ronin binding motif are necessary and sufficient for P clustering (A) RNA FISH with probes targeting the *Gsk3a* and *Rabac1* loci (illustrated in Figure 1F) in control and *Ronin* knockout ESCs after 4 days of tamoxifen treatment. White boxes represent the magnified areas shown at the bottom. *Gsk3a*, *Rabac1*, and an overlay of both signals are shown from left to right. (B) Quantification of RNA FISH analyses as shown in (A). Boxplots show the distance between the closest green and red signals per cell measured after detecting indicated loci that are looped by Ronin and orthogonal controls that have been described previously to interact but are not bound by Ronin (looped in a Ronin-independent fashion). n = 208, 208, 78, 44, 61, 76, 97, 101, 74, 108, 110, 108; p = 6.8 × 10^15^, 0.00329, 0.04587, 2.8 × 10^7^, 0.20614, 0.58125 by t test from left to right). The distance between the TSSs of the genes is reported under the gene names. (C) 3C-PCR analyses of Ronin-looped Ps in comparison with genomic sites that are known to interact (e.g., *Myc* and *Pvt* Ps, *Phc1* E and its P) but are not bound by Ronin. Representative gel images (bottom) and quantifications as boxplots (top) are shown. n = 3; p = 0.02211, 0.03567, 0.01146, 0.03557, 0.91768, and 0.52944 by t test from left to right. Note that the direct interaction between *Gpbp1l1* and *Toe1* was predicted to occur when the *Mmachc* P interacts with both Ps in the same cell. The distance between the TSSs of the genes is reported under the gene names. (D) Illustration of the strategy to test whether the RBM is necessary and sufficient for P looping using CRISPR-Cas9 deletion of one RBM in interactions that are mediated by a single RBM in one of two interacting loci. (E) Illustration of the *Snx14/Zfp949* interactions (top) and the *Snx14* alleles (bottom) that were targeted by CRISPR-Cas9 in comparison with the wild-type (WT) allele. (F) Ronin chromatin immunoprecipitation results of the *Snx14* locus in wildtype cells and cells after CRISPR-Cas9 targeted deletion of the RBM in the *Snx14* P (left). Data are represented as mean ± SD; n = 4, p = 0.00828 by t test) and boxplots showing the distance between the closest green and red signals per cell measured after RNA FISH detecting the *Snx14 and Zfp949* loci (right) in wildtype control cells (n = 65) or cells with RBM deletion (delRBM). n = 82;p = 1.5×10^7^ by t test. Related to Figures S4-S5.

We next used multiple experimental approaches to confirm the Ronin-associated promoter-promoter interactions and determine whether they are dependent on Ronin by comparing conditional *Ronin* knockout cells after 4 days of tamoxifen treatment with control cells (Figures S5A and S5B). We first used RNA fluorescence in situ hybridization (FISH) and found that all tested Ronin-mediated promoter-promoter interactions were visible as colocalizing FISH signals that showed less colocalization after *Ronin* knockout, while DNA interactions known to be Ronin independent were not significantly altered (Figures 3A and 3B; Tables S1J and S1K). Similarly, promoter-promoter interactions were confirmed by chromosome conformation capture PCR (3C-PCR), and most of the Ronin-dependent interactions were significantly reduced after conditional *Ronin* knockout (Figure 3C). To exclude possible secondary effects on DNA looping after 4 days of tamoxifen treatment, we additionally developed a temperature-sensitive Ronin mutant (F80L) and rapidly depleted the Ronin protein from cells (Figure S5C). RNA FISH with these cells confirmed the disappearance of Ronin-mediated loops seen upon genetic ablation (Figure S5D).

Next, we verified that direct binding of Ronin to DNA itself was responsible for the loops. Specifically, we used the CRISPR-Cas9 system to generate small deletions in the RBM that abolish Ronin occupancy (Figure 3D). To allow optimal RNA FISH signal detection, we selected interactions where (1) one locus interacted with only one other locus, (2) both loci showed a decent basal expression level, (3) both loci did not show drastic expression changes upon *Ronin* loss, and (4) at least one of the loci contained only one RBM, hence preventing effective Ronin loop formation after its deletion. Indeed, in each case examined, RNA FISH analyses showed a loss of promoter-promoter interaction at these sites (Figures 3E, 3F, and S5E-S5J). Together, the experimental results provide evidence that promoter-promoter looping among multiple housekeeping genes is dependent on Ronin.

**Figure 4.**
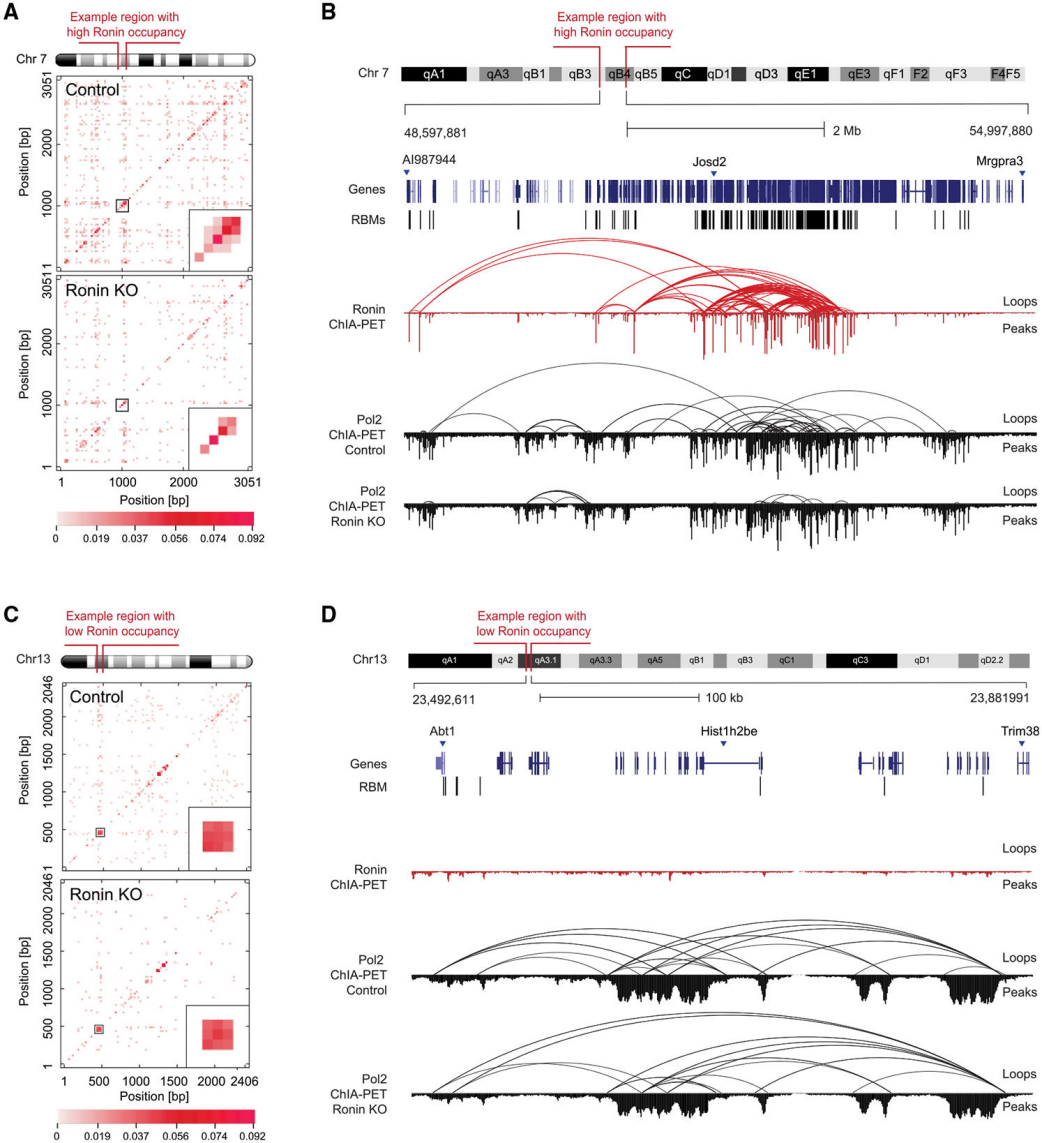
Loss of Ronin does not change RNA polymerase II (RNA Pol II) occupancy but leads to loss of P interactions between Ronin-bound genes (A) Heatmap of RNA Pol II-containing interactions between different loci on chromosome 7 (chr 7) in control (top) and *Ronin* knockout (KO) ESCs (bottom). Shown are the merged results from two ChIA-PET experiments per condition. The inset in the bottom right corner shows a magnification of the boxed region. (B) Close-up illustration of a chromosomal region (corresponding to the boxed region in B) that is highly occupied by Ronin, showing RNA Pol II occupancy and interactions in control and *Ronin* KO cells and the overlap with Ronin peaks and loops in WT cells. Shown are the merged results from two ChIA-PET experiments per condition. Arc plots include interactions that start and end in the chromosomal region shown. Ctrl, control; n.s., not significant. (C) Heatmap of polymerase interactions between different loci on chr13 in Ctrl (top) and Ronin KO cells (bottom). The inset in the bottom right corner shows a magnification of the boxed region. (D) Close-up illustration of a chromosomal region (corresponding to the boxed region in C) with low Ronin occupancy and Ronin-independent loops that remain unchanged after *Ronin* KO. Related to Figure S6.

To further confirm that Ronin-associated promoter-promoter interactions are dependent on Ronin, we conducted ChIA-PET with antibodies directed against RNA polymerase II (RNA Pol II) in control and *Ronin* knockout cells after 4 days of tamoxifen treatment (Table S2). RNA Pol II was used in this experiment because it is known to accumulate at promoter-proximal pause sites at all active genes, even when gene transcription does not occur.^69^ We found that, in control ESCs, 95% of Ronin targets (including 96% of the Ronin-looped genes) were also RNA Pol II-occupied genes, and 81% of genes associated with RNA Pol II interactions were Ronin target genes (including 75% of Ronin-looped genes) (Figures S6A). While RNA Pol II occupancy did not change (Figure S6B) (98.5% of RNA Pol II-bound genes were shared in control and knockout cells; Figure S6C), the DNA loop interaction frequency among promoters occupied by RNA Pol II dropped substantially from 11,598 to 4,930 after *Ronin* knockout (Figure S6D; Table S2A). As expected, the most drastic changes occurred in regions that were bound and looped by Ronin in control cells (Figures 4A and 4B). In regions that were not highly occupied by Ronin, loop formation remained mostly unchanged (Figures 4C and 4D). The drop in DNA interactions reflected a complex rearrangement of interactions, with only 7.2% of interactions that involved the same anchors being retained, contributing to 17% of the remaining anchors (Table S2F). This observation is not surprising because DNA looping factors other than Ronin, such as other members of the THAP family, must have been engaged to ensure proper housekeeping gene promoter clustering. While 52% of genes that were looped by Ronin in wild-type cells were still involved in interactions (Figure S6E) after *Ronin* knockout, the PET count of approximately 81% of genes involved in RNA Pol II-associated DNA interactions (including 75% of Ronin-bound sites) dropped significantly (Figures S6F and S6G). Among those Ronin-looped genes were the ones with the highest initial RNA Pol II-PET counts, which lost an average of 46% of their interactions (Figure S6G). Together, these data suggest that Ronin arranges a significant portion of the housekeeping promoter-promoter loops in the ESC genome.

### Proper transcription of occupied housekeeping genes depends on Ronin

**Figure 5.**
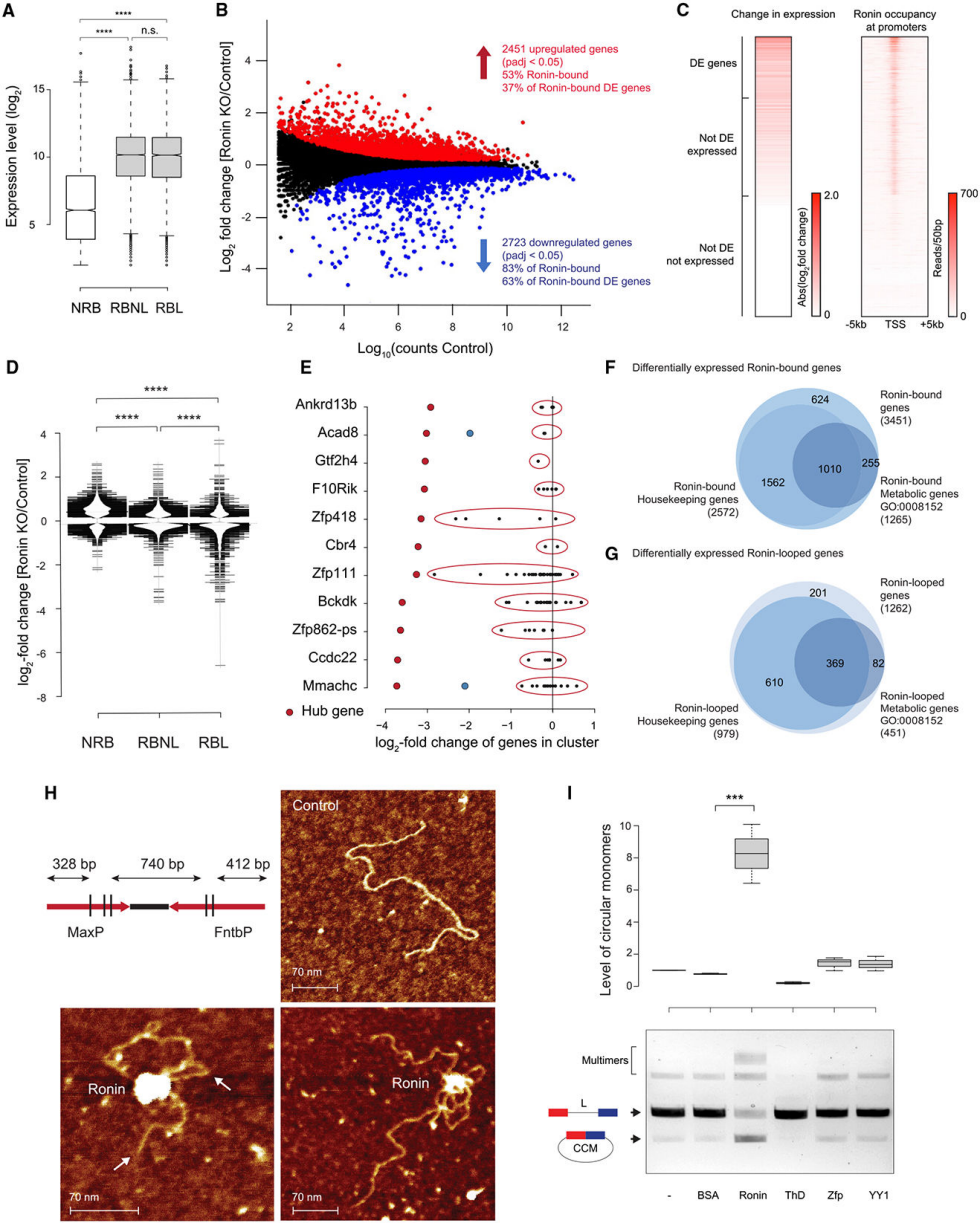
Ronin regulates transcription of housekeeping genes and evolved from an ancient transposon to loop DNA (A) Expression level of genes (with more than 3 reads) that are not bound by Ronin (NRB; n = 6,149), bound by Ronin but not looped by Ronin (RBNL; 0–2 Ronin PETs, n = 7,089), or Ronin bound and looped (RBL/anchors; ≥ 3 Ronin PETs, n = 3,104). p = 0 (NRB/RBNL), 0 (NRB/RBL), 0.99328 (RBNL/RBL) by t test. (B) Change in gene expression (log_2_ fold change) in *Ronin* KO cells after 4 days of tamoxifen treatment for all genes plotted against expression in Ctrl cells. Genes that displayed significant changes in expression (false discovery rate [FDR]-adjusted p < 0.05) are colored, with upregulated genes plotted in red and downregulated genes plotted in blue. (C) Heatmaps displaying the gene expression changes in *Ronin* KO cells (left) and Ronin occupancy in a ±5-kb region centered on the transcription start site (TSS) of each gene (right). Each row represents a single gene, and genes are ranked by their adjusted p value for change in expression in *Ronin* KO cells compared with Ctrl cells. (D) Changes in gene expression between *Ronin* KO and Ctrl cells, reported as absolute shrunken log_2_ fold change for genes that are NRB, RBNL (0–2 PETs), and RBL (≥3 PETs). p = 2.3e–109 (NRB/RBNL), 1.8^70^ (NRB/RBL), and 1.2^24^ (RBNL/RBL), as determined by t test. (E) Fold change of genes within individual clusters that are related to the hub genes (red circles, named on the y axis) that are downregulated the most within each cluster after *Ronin* KO. Blue circles represent genes that are controlled by the same P (bilateral) as the hub gene. (F) Venn diagram of differentially expressed protein-coding genes that are bound by Ronin, housekeeping genes, and metabolic genes. Only protein-coding genes were considered for the overlap for consistency. Statistical enrichment for housekeeping genes (p = 1.7e–133) and metabolic genes (p = 7.1^8^) was determined by hypergeometric testing. (G) Same as (C) but only considering genes whose Ps are involved in P-P interactions. Statistical enrichment for housekeeping genes (p = 4.0^37^) and metabolic genes (p = 0.01) was determined by hypergeometric testing. (H) AFM images of linear substrate DNA (top right) that contains two Ronin-bound Ps with several RBMs (black bars) that are separated by a short DNA fragment (top left). In the presence of recombinant Ronin protein, we observed circular monomers held together by Ronin binding to the RBMs at the opposite ends of the DNA (bottom left)(white arrows indicate the 328- and 412-bp overhangs) and Ronin protein bound to multiple DNA molecules in *trans* (bottom right). FntbP, *Fntb* P. (I) Representative image (top) and quantification (bottom) of gel shift circularization assays using a linearized 5-kbp substrate harboring two Ps with RBMs in the absence or presence of different recombinant proteins. YY1, which is known to loop DNA but cannot bind the DNA template used in this experiment, served as an additional negative Ctrl. Data are represented as mean ± SD; n = 3, p = 0.00028 by t test. CCM, covalently closed circular monomer; L, linear substrate; ThD, Thap domain (amino acids 1–80 of the Ronin protein); Zfp, Zfp143. Related to Figure S7 and Tables S1 and S3.

To pursue the hypothesis that Ronin influences transcription of the clusters of housekeeping genes that it occupies, we performed RNA-seq analyses with control and *Ronin* knockout cells after 4 days of tamoxifen treatment (Table S3A). As expected from the housekeeping nature of Ronin targets, we found that 67% of all expressed genes (those with more than 3 reads) were Ronin targets (Figure S7A); moreover, independent of their involvement in interactions, these genes were expressed at a significantly higher level than those that were not bound by Ronin (Figure 5A). We found that approximately 5,000 genes showed differential expression, with a similar proportion increasing (47%) and decreasing (53%) in expression levels (Figure 5B). Ronin was bound to approximately 70% of these differentially expressed genes (Figure S7B), of which 63% were downregulated and 37% were upregulated (Figures 5B and 5C; Tables S3B and S3C). Secondary and indirect effects that accompany Ronin knockout may result in the differential expression of the remaining 30% genes.

Ronin loss leads to upregulation and downregulation of target genes, which is similar to results obtained with YY1,^14,17^ a factor that facilitates loop formation and whose loss causes increases and decreases in expression of the genes it occupies.^71^ Loss of Ronin looping may have indirect effects on cell survival and housekeeping demands, potentially through Ronin-independent DNA interactions or binding of other transcription factors (e.g., Zfp143). Hence, we analyzed the gene expression changes in more detail; comparison of fold change differences (Figure 5D) and gene set analyses (Figure S7C) confirmed that Ronin targets were overall downregulated, with the most drastic effects in Ronin target genes that we had identified to be involved in interactions (associated with anchors with ≥3 PETs) in wild-type cells. Not surprisingly, the differential expression was correlated with the number of RBMs present in their anchors (Figure S7D). Interestingly, not all genes in any given promoter cluster were affected to the same extent. When we analyzed some of the most highly downregulated genes, we found that they belonged to distinct clusters in which they served as “hub” genes (highly connected anchors with the most RBMs within a cluster). The other genes found within the same cluster showed different degrees of expression and regulation, usually much less than those of the corresponding hub gene (Figure 5E). Importantly, changes in RNA Pol II-containing interactions (as expected from their high overlap with Ronin targets and Ronin-looped genes; Figures S6A) directly correlated with the *Ronin* knockout-induced gene expression changes. Genes that lost RNA Pol II-associated interactions (PETs) upon *Ronin* knockout were overall downregulated, while those that gained RNA Pol II-associated loops were upregulated (Figures S7E). Finally, consistent with the observation that Ronin is bound to promoters of most housekeeping and many metabolic genes, genes whose expression was altered after Ronin loss showed a significant overlap with housekeeping and metabolic genes (Figures 5F and 5G; Tables S3B and S3C). Overall, our data suggest that Ronin arranges housekeeping genes in promoter-promoter clusters to regulate transcription, although loss of Ronin does not necessarily lead to co-regulation of all genes that belong to the same cluster.

### Ronin stimulates DNA loop formation *in vitro*

We have so far described evidence showing that Ronin binds to the core promoters of clustered housekeeping genes, that looping of these promoters into an assembly is dependent on Ronin, and that the expression of many of these genes is dysregulated after loss of Ronin. We next turned to the question of whether Ronin is a tethering factor that by itself can physically connect DNA sequences containing its binding motif. To address this question, we first performed atomic force microscopy (AFM). Using a DNA substrate that harbors asymmetrically long overhangs flanking RBMs and purified, recombinant Ronin, we were readily able to visualize circular monomers as well as multiple DNA molecules bound to Ronin protein (Figure 5H). To support the AFM data, which are qualitative in nature, we next used *in vitro* DNA circularization assays to quantify Ronin’s looping ability. Consistent with our hypothesis, these experiments showed that Ronin stimulates formation of circular DNA monomers and multimers (Figure 5I). We quantified the most dominant monomeric product and confirmed its identity by restriction digestion (Figure S7F). In contrast, circular DNA failed to form in the presence of (1) mutant Ronin protein that included the DNA-binding domain but lacked the dimerization domain; (2) Zfp143, a Ronin antagonist shown previously to bind the Ronin target sequence but lacking a dimerization domain;^53,72-75^ or (3) the enhancer-promoter looping factor YY1.^17^ Ronin also did not form loops or enhance circularization in the presence of short specific competitor DNA containing the RBM, whereas a nonspecific competitor had no effect (Figure S7G). Together, these experiments verify that Ronin can bring two DNA elements together through binding of DNA elements *in vitro* and provide a mechanism to explain how this activity may facilitate promoter-promoter interactions *in vivo*.

### Ronin-associated biomolecular condensates

**Figure 6.**
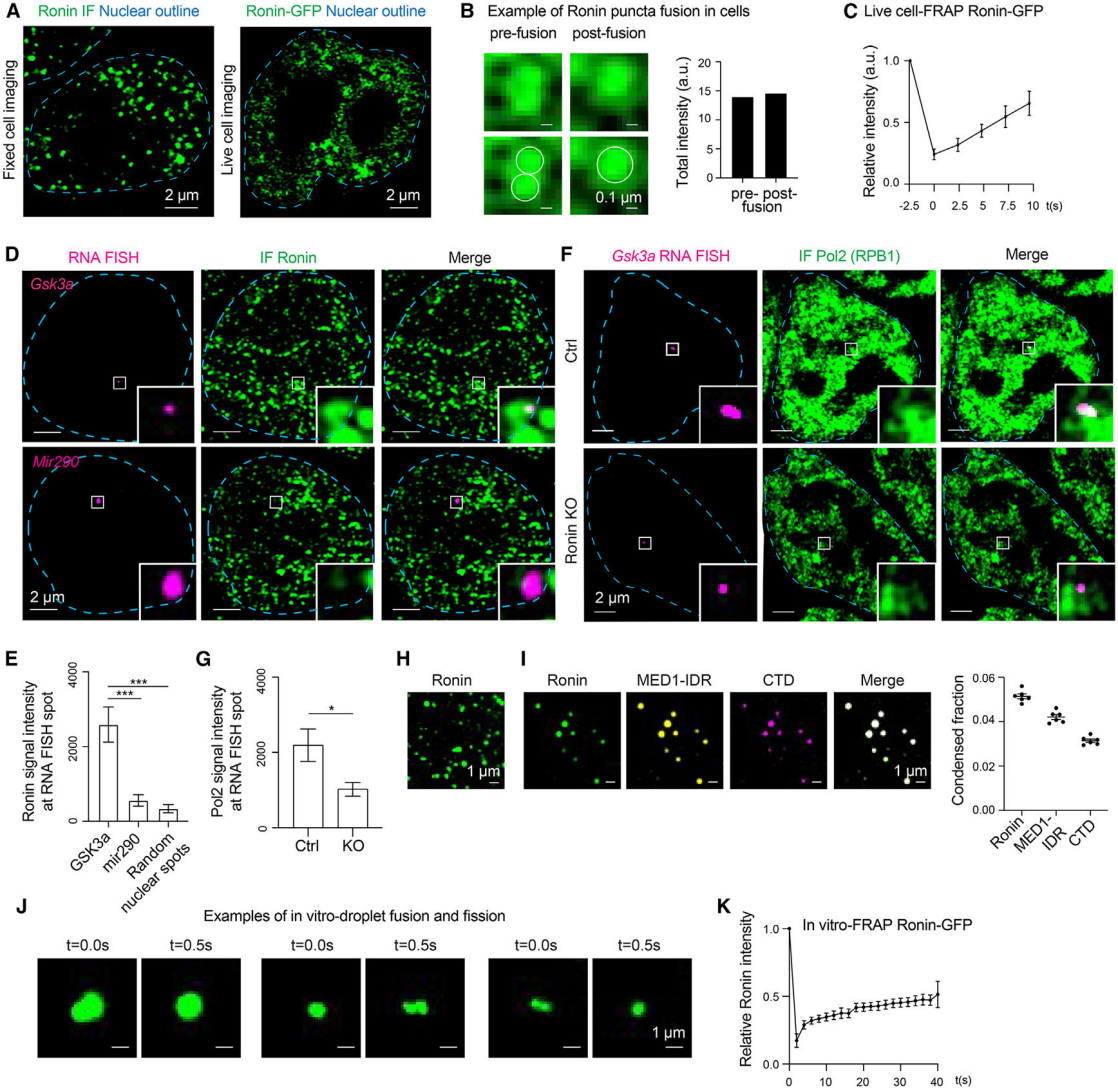
Ronin-containing condensates at Ps of housekeeping genes (A) Representative immunofluorescence image after detection of Ronin (green) (left) and live-cell immunofluorescence image (right) of Ronin-GFP-expressing mESCs. Dashed blue lines represent the nuclear outline. (B) Representative images of Ronin-GFP puncta *in vivo* before and after fusion (top left) and quantification of total intensity (bottom left and right) expressed in arbitrary units (a.u.). The intensity before fusion reflects the sum of both puncta. The images were taken 0.4 s apart. (C) Quantification of the fluorescence recovery after photobleaching (FRAP) experiment of Ronin-GFP puncta *in vivo*. a.u. are represented as mean ± SD; n = 5, p = 0.005 (0 s/10 s) by t test. (D) Immunofluorescence-RNA FISH images for Ronin (immunofluorescence [IF], green) and *Gsk3a* (RNA FISH, magenta) (top) or Ronin (IF, green) and *mir290* (RNA FISH, magenta) as a negative Ctrl (bottom). Dashed blue lines represent the nuclear outline. An approximately 0.2-nm-thick nuclear section is shown. (E) Quantification of Ronin IF signals at RNA FISH puncta or Radom nuclear spots in Ctrl cells. Data are represented as mean ± SEM; p < 0.001 by t test. (F) Images of RNA Pol II (RPB1, green) and nascent RNA of *Gsk3a* (magenta) in Ctrl and *Ronin* KO cells. Dashed blue lines represent the nuclear outline. An approximately 0.2-nm-thick nuclear section is shown. (G) Quantification of RNA Pol II (RPB1) signal intensity at *Gsk3a* transcription sites in Ctrl and *Ronin* KO cells(right). Data are represented as mean ± SEM; n = 9, p = 0.03 by t test. (H) Images of Ronin *in vitro* droplets. (I) Images of Ronin *in vitro* droplets (green), MED1-IDR (yellow), CTD (magenta) (left) and their quantification, shown as dot plot (right). Data are represented as mean ± SEM; n = 6. (J) Representative images of Ronin *in vitro* droplets undergoing fusion and fission. (K) Quantification of FRAP before and after bleaching of Ronin-GFP *in vitro* droplets. Data are represented as mean ± SD; n = 3, p = 0.05 by t test.

The clustering of multiple enhancers and their interaction with the promoters of cell identify genes is associated with formation of assemblies of transcriptional components that have been called hubs, clusters, and condensates.^26-29,31,33-37,39,40,76,77^ Condensates are dynamic assemblies of large numbers of molecules and can be observed as puncta in cells when the protein of interest is fused to green fluorescent protein (GFP). Immunofluorescence and live-cell imaging revealed that Ronin does occur in puncta in the nuclei of mESCs (Figure 6A). These puncta had features of liquid-like biomolecular condensates; they showed evidence of fusion (Figure 6B) and rapid molecular exchange kinetics (Figure 6C). To determine whether Ronin puncta occur at housekeeping genes, immunofluorescence combined with RNA FISH was used at the *Gsk3a* locus, and the results confirmed that Ronin formed puncta at this locus (Figures 6D and 6E). When this experiment was conducted for the cell identity gene *Mir290*, which is not looped by Ronin, there was significantly less Ronin incorporation into puncta at this locus relative to that observed with the *Gsk3a* locus (Figures 6D and 6E). These results are consistent with the possibility that Ronin forms promoter clusters at active housekeeping genes that assemble sufficient DNA and protein molecules to form biomolecular condensates.

To test whether the Ronin-associated biomolecular condensates that occur at housekeeping genes are dependent on Ronin, we used confocal imaging of RNA Pol II in control and *Ronin* knockout cells after 4 days of tamoxifen treatment. As expected, large numbers of RNA Pol II molecules were found in transcriptional condensates in control cells, which allowed monitoring of the RNA Pol II signal at housekeeping gene loci of interest (Figure 6F, top). When we compared the RNA Pol II signal intensity in *Ronin* knockout cells, we not only found a 2-fold reduction at the Ronin-looped Gsk3a locus, visualized by RNA FISH (Figure 6F, bottom), but a general drop in intensity of the RNA Pol II signal in condensates at this locus (Figures 6F and 6G). This reduced immunofluorescence signal of RNA Pol II in condensates is likely due to the diminished concentration of RNA Pol II in transcriptional condensates in *Ronin* knockout cells because RNA Pol II occupancy at genomic loci is similar in control and *Ronin* knockout cells (Figures 4 and S6B).

Proteins that contribute to biomolecular condensates in nuclei can often form liquid-like droplets *in vitro*,^37^ and we found that purified Ronin formed droplets *in vitro* (Figure 6H). Previous studies have shown that transcription factors that promote condensate formation can form heterotypic droplets *in vitro* with MED1-IDR (the intrinsically disordered region of the large subunit of the Mediator coactivator) and the CTD of RNA Pol II.^27,78^ Ronin also formed droplets that concentrated these components of transcriptional condensates (Figure 6I), and the Ronin droplets showed evidence of fusion and fission (Figure 6J) and a rapid exchange kinetic (Figure 6K), properties expected for liquid-like condensates. We infer from these results that Ronin molecules can form liquid-like condensates *in vitro* that incorporate key components of transcriptional condensates.

## DISCUSSION

**Figure 7.**
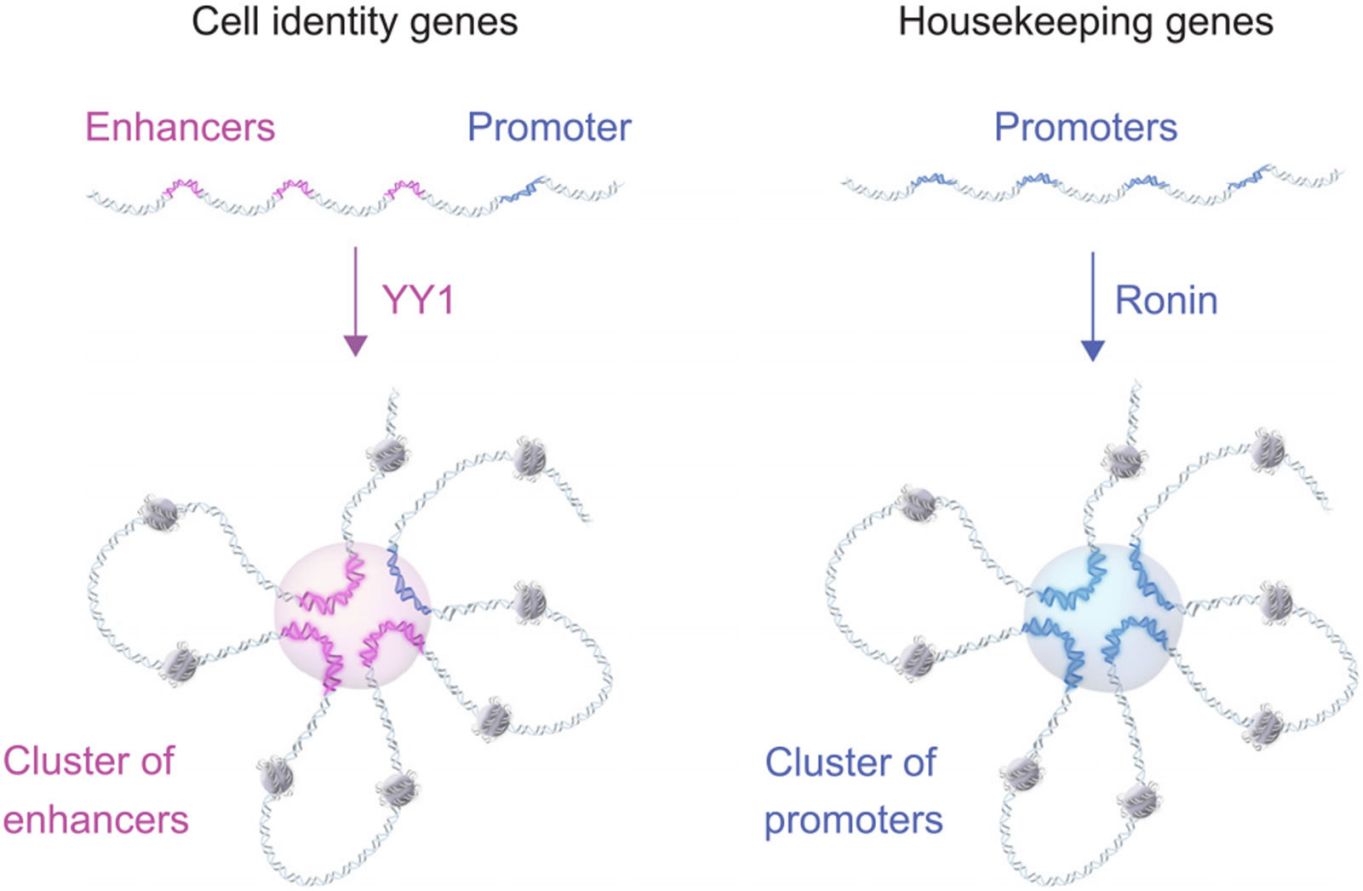
Models of regulatory element clustering We propose that control of cell identity and housekeeping genes employs a common strategy. To regulate cell identity genes, multiple Es loop to a P, facilitated by bundling factors such as YY1, and concentrate the assembled transcription apparatus (left). In the case of housekeeping genes, multiple Ps are clustered, facilitated by Ronin, and concentrate the assembled transcription apparatus at these genes (right).

This study draws attention to an unconventional model of housekeeping gene regulation whereby Ronin, a factor previously implicated in cellular growth control, bundles gene promoters, thus promoting formation of assemblies that facilitate proper regulation of genes within the clusters. This model of regulation of housekeeping gene clusters is analogous to that proposed to compartmentalize the transcription apparatus at the clustered enhancers of genes key to cell identity (Figure 7). The promoters of clustered housekeeping genes are bundled by Ronin, which multimerizes to bring these promoter elements together, whereas the clustered enhancers of cell identity genes are bundled by factors such as YY1, which multimerizes to bring the enhancers and promoter elements together.^14,17^ In both cases, there are many additional factors and components that contribute to proper gene regulation, but the concept that gene regulation is linked to chromosome architecture is now extended to housekeeping gene clusters through promoter-promoter interaction.

Our results contribute to the emerging view that specific transcription factors provide tethering functions that contribute to proper genome architecture because of their ability to bind DNA and to bridge DNA sequences through multimerization. These factors include, but are not limited to, YY1, CTCF, BORIS, NANOG, KLF4 and ThPOK in mammalian cells and Trl/GAGA in *Drosophila*.^13-17,30,38,79-81^ Proper genome organization is linked to gene control at multiple levels. Tethering of multiple regulatory sites is necessary for proper transcriptional dynamics,^13^ whereas tethering of the boundaries of topologically associating domains (TADs) can prevent spurious interactions with regulatory elements located in neighboring TADs.^2,3,8,10,12,13,22,82-84^

Ronin’s architectural function evolved from an ancestor of the P element transposon, a domestication process that our data suggest might have been driven by the transposon’s proclivity for binding to the promoters of metabolic genes and its ability to loop DNA. In fact, the P element moves within the germline at a time when most expressed genes are metabolic genes that are very close to each other in the genome.^46,85^ There is a striking commonality between Ronin’s structure and behavior and that of other members of the Thap-domain-containing protein family,^59^ which likely oversee related genetic programs and configure different kinds of gene clusters. Thus, it is easy to imagine that different THAPs might shape somewhat different sets of housekeeping genes, depending on different cellular contexts (e.g., highly prolific vs. quiescent cells). It is possible that other THAPs similar to Ronin might play a similar role in creating connections between gene promoters, and possibly between enhancers and promoters, and could compensate and prevent changes in how Ronin target genes are expressed.

The model we describe here for regulation of housekeeping genes may lead to insights into the mechanisms by which metabolic alterations contribute to disease. Over 2,000 metabolic genes have been shown to undergo alterations in cancer cells, and metabolic reprogramming fuels cancer cell growth and metabolism.^86,87^ The insights into housekeeping gene regulation described here should provide the foundation to decipher mechanisms by which some metabolic genes are reprogrammed in tumor cells and perhaps lead to therapeutic hypotheses not discussed so far.

### Limitations of the study

While this study focuses on mESCs, in which Ronin’s function has been best characterized,^50,61,62^ Ronin is generally expressed in diverse cell types, and DNA interaction data from MEFs and resting B cells indicate that clustered promoters occur in a manner similar to that observed in ESCs,^49^ suggesting that Ronin-mediated promoter clustering is a general mechanism to coordinate efficient expression of housekeeping genes. Genome-wide experiments revealed that many promoter-promoter interactions among Ronin-bound housekeeping genes are lost in *Ronin* knockout cells, suggesting that Ronin is important for formation of these interactions. Given that the different experimental techniques used in our study have their own caveats, multiple orthogonal approaches were used to test our hypothesis. To validate our genome-wide findings at target gene loci, 3C-qPCR and imaging experiments were performed. While 3C-qPCR allows high-resolution investigation of promoter-promoter contacts, imaging experiments suffer from lower resolution because of the limitation of the microscopy tools, and the high degree of colocalization observed by imaging could be due to low resolution. Additionally, 4 days of tamoxifen treatment were required for proper Ronin downregulation in *Ronin* knockout cells, and therefore it is possible that other indirect mechanisms could contribute to the loss of promoter-promoter contacts. To address this limitation, a temperature-sensitive Ronin mutant was used, allowing us to assess the immediate changes in chromatin interactions only 4 h after switching to a non-permissive temperature. Also, while our data suggest that the attenuated incorporation of RNA Pol II into condensates is caused by loss of Ronin interactions between RNA Pol II-occupied promoters, *Ronin* knockout could indirectly affect RNA Pol II-promoter interactions that are established by other looping factors. Additionally, the resolution of each ChIA-PET experiment is highly dependent on the antibody and experimental variation, and data processing could influence the identification of DNA interactions. Hence, we might underestimate the DNA interactions between promoters that we detected in the RNA Pol II and Ronin ChIA-PET experiments, and the definition of bound (targets) versus looped (anchors) genes might not be as static. Finally, it is important to note that spike-ins were not included in the ChIA-PET experiments. While they are commonly used in comparative chromatin immunoprecipitation sequencing (ChIP-seq) experiments, to our knowledge, there is no accepted published protocol in the field for the use of spike-ins in ChIA-PET experiments. The goal of the RNA Pol II ChIA-PET experiment was to determine the changes in DNA loop formation as a consequence of *Ronin* knockout and not as a consequence of changes in RNA Pol II occupancy. Spike-in controls might have interfered with this analysis because (1) use of spike-ins is known to introduce experimental variation,^88^ and (2) species cross-reactivity of antibodies, affecting subsequent data interpretation, is a known caveat of the use of spike-ins.

## STAR★METHODS

### RESOURCE AVAILABILITY

#### Lead contact

Further information and requests for resources and reagents should be directed to and will be fulfilled by the lead contact, Thomas P. Zwaka (thomas.zwaka@mssm.edu).

#### Materials availability

All unique reagents in this study are available from the lead contact and will be provided upon request. There are restrictions to the availability of frozen cell vials due to the number of frozen stocks available.

#### Data and code availability

- All high-throughput sequencing data have been deposited at GEO (GSE136145) and are publicly available. This paper analyzes existing, publicly available data. These accession numbers for the datasets are listed in the key resources table.
- This paper does not report any original code. All software packages and their accessibility are described in the STAR Methods sections.
- Any additional information required to reanalyze the data reported in this paper is available from the lead contact upon request.

### EXPERIMENTAL MODEL AND SUBJECT DETAILS

#### Cell lines

R1 (ZCL1000), inducible *Ronin* knockout (CreERT2; Ronin^flox/flox^, ZCL1032), D3-Ronin-GFP (ZCL1029)^61^ and control (Ronin^flox/flox^, ZCL1040) embryonic stem (ES) cells^107^ and their derivatives were cultured on irradiated mouse embryonic fibroblasts (MEFs) or in culture dishes coated with 0.1% gelatin (Sigma, G1890) with Dulbecco’s Modified Eagle’s Medium (DMEM) containing high glucose, GlutaMax and Pyruvate (Invitrogen, 10569010) supplemented with 2 mM glutamine (Invitrogen), 100 nM MEM Non-Essential Amino Acids (Invitrogen), 100 μM 2-mercaptoethanol (Fluka), 1000 U/ml Leukemia inhibitory factor (Lif, Millipore, ESG1107) and 50 μg/mL hygromycin B (Invitrogen, 10687-010) or when applicable. Irradiated MEFs (GlobalStem, GSC-6001G or DR4, GlobalStem, GSC-6204G) were cultured in DMEM (Invitrogen) supplemented with 10% fetal bovine serum (FBS; Sigma, F4135), 2 mM glutamine (Invitrogen), 100 nM MEM Non-Essential Amino Acids (Invitrogen) and 100 μM 2-mercaptoethanol (Fluka).

#### Mice

All experimental procedures and protocols of animal research were approved by the Institutional Animal Care and Use Committee of Baylor College of Medicine. Inducible Ronin CreERT2; Ronin^flox/f**lox**^ (ZCL1032) were isolated from embryos at day 3.5 after first crossing Rosa26 ^Ert2-Cre/+78^ with Ronin^loxP/*loxP*^ animals and then with Ronin^loxP/−^ animals from Ronin^loxP/*loxP*^ crosses with Ronin^+/−^.^61^ ES cells were derived from the blastocysts of 3- to 5-week-old female mice that had been induced to super-ovulate and then mated with stud males. Noon of the day that a vaginal plug was observed was considered embryonic day 0.5 (E0.5). At E3.5, blastocysts were flushed from the female uterine horns, and each blastocyst was seeded into a single well of a 4-well dish containing irradiated MEF feeder cells and standard ES derivation medium (Knockout DMEM, with 2mM L-glutamine, 100 μM nonessential amino acids, 100 μM 2-mercaptoethanol, 2 × 10^3^ U/ml Lif, and 20% Knockout Serum Replacement) at 37°C with 5% CO_2_ for 5 days. The cultured cells were dissociated and passaged in standard mouse ES culture medium until pluripotent ES cells emerged.

### METHOD DETAILS

#### Plasmid construction

To generate pGL3-PMax-Luc-PFntb (pTZ2073), an 852-bp region around the transcription start site of *Fntb* (−277 to +575 bp) was amplified from mouse genomic DNA (R1 mES cells) with primers Z20-127 (pFntb-F1-SalI, AGT CGT CGA CAT CGC AAC TAC TGC ATT AC) and Z20-128 (pFntb-R1-SalI, CAT GGT CGA CGA AAC TGC GAA CAT GTG AAG) and cloned into the SalI site of pGL3-Basic-MaxPro (pTZ1841).^50^ The resulting plasmid contained part of the *Max* promoter (from −903 to 125 bp around the transcription start site) separated from the *Fntb* promoter (in antisense orientation) by the luciferase gene. pSMaxFntbAs (pTZ3023), was created by deleting the Luciferase gene from pTZ2073 to shorten the sequence between the *Max* and *Fntb* promoters. pTZ2073 was cut with XbaI (NEB, R0145S) and NheI (NEB, R0131L) and the 5383 bp fragment was religated after gel electrophoresis and purification with the Qiagen Gel Extraction Kit (28704) following the manufacturer’s protocol.

Plasmids containing the guide RNA needed to target the Ronin-binding motifs (RBMs) in the promoters of *Snx14*, *4930430F08Rik* and *Ubxn4* for deletion by CRISPR-Cas9 genome editing were generated according to Ran et al.^84^ Briefly, single-stranded guide RNA oligos were annealed and cloned into the Bbs1 site of pSpCas9(BB)-2A-GFP.^84^ The sequences of the single-stranded oligos were as follows: Snx14 gRNA: Z10-732 (Snx14Crispr-T), CAC CGG TAT GGA CTA CAT TTC CCA G and Z10-733: Snx14Crispr-B), AAA CCT GGG AAA TGT AGT CCA TAC C; 4930430F08Rik gRNA: Z10-734 (F08Rik Crispr-T), CAC CGG GAA TTG TAG TGC GAC CGC G and Z10-735 (F08RikCrispr-B), AAA CCG CGG TCG CAC TAC AAT TCC C; Ubxn4 gRNA: Z10-736 (Ubxn4Crispr-T), CAC CGC TGG GAA TTG TAG TCT TCC G and Z10-737 (Ubxn4Crispr-B), AAA CCG GAA GAC TAC AAT TCC CAG C. The resulting plasmids were designated pCas9-gSnx14 (pTZ3019), pCas9-gRikF08 (pTZ3020) and pCas9-gUbxn4 (pTZ3021), respectively.

#### Cell culture, generation of cell lines, induction of *ronin* knockout and proliferation assay

To induce *Ronin* knockout, 100,000 cells/10 cm^2^ of inducible *Ronin* knockout (CreERT2; Ronin^flox/flox^, ZCL1032 were plated on gelatin-coated cell culture dishes. After 6 h, tamoxifen (Sigma, T176) was added at a final concentration of 0.25 μM (dissolved in ethanol). Control cells were treated with ethanol alone. The cells were fed for 4 days with medium containing 0.25 μM tamoxifen or ethanol. For the growth curve, cells were then split at a density of 100,000 cells/10 cm^2^ and cultured further in standard mouse ES cell medium. Cells were counted with a ViCell XR2.03 (Beckman Coulter) cell counter.

Cell lines with deletions of the RBM in *Snx14*, *4930430F08Rik* or *Ubxn4* promoters were generated with the CRISPR-Cas9 genome editing system.^84^ ES cells (R1) were transduced with pCas9-gSnx14 (pTZ3019), pCas9-gRikF08 (pTZ3020) or pCas9-gUbxn4 (pTZ3021), respectively (see above), using Lipofectamine2000 as recommended by the manufacturer. At 24 h post-transfection, EGFP-positive cells were sorted by FACS and plated on MEFs at different densities. After 10 days, single colonies were picked and expanded. To check the allele status, DNA was isolated with the Blood and Tissue Kit (Qiagen, 69506) and used as template in PCRs that were performed with GoTaq green (Promega) and the following oligos: Snx14 (698 bp): Z10-749 (Snx14-SR), GAG CAG GAT CTT GCT TGT CTC AGC and Z10-748 (Snx14-SF), CCC AGC TTC ACA GCT AGA CTC AGC; 4930430F08Rik (803 bp): Z10-744 (F08Rik-SF), AGA TAC AAG AAG CTG GAG GTC AGG and Z10-745 (F08Rik-SR), CAA AAA ACA AAC TGT ACA CTG CGT GG; Ubxn4 (859 bp): Z10-740 (Ubxn4-SF), AGC TGT CCA TCT CAT ACA GGC AGG and Z10-741 (Ubxn4-SR), CTC ACT CGA CGC TAA CGA GAA TCC. The expected PCR fragment sizes correspond to the respective wildtype alleles. The PCR products were purified (PCR purification kit, Qiagen) and cloned into pCR2.1-Topo with the Topo TA cloning kit (Invitrogen, K4500J10), as recommended by the manufacturer. Several colonies were used to inoculate fluid cultures, plasmid DNA was isolated (QIAprep Spin Miniprep Kit, 27106) and the sequences of both alleles were determined using universal sequencing primers M13R (GTA AAA CGA CGG CCA GT) and M13R-pUC (CAG GAA ACA GCT ATG AC).

#### Chromatin interaction analysis by paired-end tag sequencing (ChIA-PET)

High-throughput sequencing after chromatin immunoprecipitation was carried out for Ronin and Pol2 using the ChIA-PET method,^22^ a modified version of a previous ChIA-PET protocol.^64^ Mouse ES cells (500 million cells, grown to ~80% confluency) were crosslinked at room temperature with 1.5 mM ethylene glycol bis(succinimidyl succinate) (EGS) for 30 min, treated with 1% formaldehyde for 10 min, and then neutralized with 125 mM glycine. Crosslinked cells were washed three times with ice-cold PBS, snap-frozen in liquid nitrogen, and stored at −80°C before further processing. Protein G Dynabeads (250 μL; Life Technologies, 10004D) were blocked with 0.5% BSA (w/v) in PBS and pre-incubated with 25 μg of anti-Ronin (clone P56-507, Becton Dickinson, 562548) or anti-RNAPII (Pol2, Clone 8WG16, Ab817, Abcam) antibodies.^108^ Nuclei were isolated^109^ and sonicated on ice in lysis buffer (20 mM Tris-HCl pH 8.0, 150 mM NaCl, 2 mM EDTA, pH 8.0, 0.1% SDS, 1% Triton X-100) using a Sonic Dismembrator (Fisher Scientific; power setting 5 for 14 cycles of 30 s each with 60 s between cycles). Sonicated lysates were cleared once by centrifugation and incubated overnight at 4°C in the presence of the antibody-bound beads. Protein-DNA complexes were sequentially washed with buffer A (50 mM HEPES-KOH, pH 7.9, 140 mM NaCl, 1 mM EDTA, pH 8.0, 0.1% Na-Deoxycholate, 1% Triton X-100, 0.1% SDS), buffer B (50 mM HEPES-KOH, pH 7.9, 500 mM NaCl, 1 mM EDTA, pH 8.0, 0.1% Na-Deoxycholate, 1% Triton X-100, 0.1% SDS), buffer C (20 mM Tris-HCl, pH 8.0, 250 mM LiCl, 1 mM EDTA, pH 8.0, 0.5% Na-Deoxycholate, 0.5% IGEPAL C-630, 0.1% SDS) and buffer D (TE with 50 mM NaCl). ChIP-Seq libraries were prepared using an adaptation of the Nextera Library Preparation protocol (Illumina).^22^ ChIP DNA fragments were end-repaired using T4 DNA polymerase (NEB, M0203) and A-tailed with Klenow (NEB, M0212). A biotinylated bridge linker (F:/5Phos/CGC GAT ATC/iBiodT/TAT CTG ACT; R:/5Phos/GTC AGA TAA GAT ATC GCG T) with T-overhangs was added and proximity ligation was performed overnight at 16°C in a volume of 1.5 mL. Unligated DNA was digested with exonuclease (NEB, M0262S) and lambda nuclease (NEB, M0293S). The ligated DNA was eluted in elution buffer (50 mM Tris-HCl, pH 8.0, 10 mM EDTA, 1% SDS), and then sequentially subjected to overnight crosslink reversal, RNase A (Sigma, R4642) treatment and proteinase K digestion. Phenol:chloroform:isoamyl alcohol extraction was performed followed by ethanol precipitation. The precipitated DNA (and then tagmented with a Nextera Tagmentation kit (Illumina, FC-121-1030) after resuspension in Nextera DNA resuspension buffer. The tagmented libraries were purified with Zymo DNA purification columns (Zymo, D4003) and subsequently bound to Streptavidin beads (Life Technologies, 11205D) to enrich for ligation fragments containing the biotinylated bridge linker. Fifteen cycles of polymerase chain reaction (PCR) were performed to amplify the library using standard Nextera primers (Illumina FC-121-1030). Following amplification, the PCR products were purified with AMPure beads (Beckman Coulter) and resuspended in 40 μL Buffer EB (Qiagen). The purified library was size selected (200–500 bp) with a Pippin prep machine and subjected to 100x100 paired-end sequencing on an Illumina HiSeq 2500 platform at the Genome Tech Core at the Whitehead Institute for Biomedical Research. All datasets were processed with a custom script.^64,82^ Image analysis and base calling were performed using the Solexa pipeline (Illumina). Reads were examined for the presence of at least 10 base pairs (bp) of linker sequence, and those lacking a linker were excluded from further processing. Reads containing a linker were trimmed using cutadapt (cutadapt -m 17; –a forward = ACG CGA TAT CTT ATC TGA CT, and –a reverse = AGT CAG ATA AGA TAT CGC GT; overlap = 10) (http://code.google.com/p/cutadapt/).^94^ Trimmed mate pairs were mapped independently to mm9 using Bowtie version 1.1.1 (bowtie -e70-k1-m1-v2-p4–best–strata–S).^93^ Aligned reads were paired with mates using an in-house script based on read identifiers. To remove PCR bias artifacts, reads were filtered for redundancy: Paired End Tags (PETs) with identical genomic coordinates and strand information at both ends were collapsed into a single PET. The PETs were further categorized into intrachromosomal or interchromosomal PETs. Regions of local enrichment (PET peaks) were called using MACS 1.4.2^101^ with the parameters “-p 1e-09 -no-lambda –no-model”. To identify long-range chromatin interactions, we first removed intrachromosomal PETs <4 kb in length because these PETs may reflect self-ligation of DNA ends from a single chromatin fragment in the ChIA-PET procedure.^83^ We next identified PETs that overlapped with PET peaks by at least 1 bp at both ends. These PETs were defined as putative interactions. A statistical model based upon the hypergeometric distribution was applied to identify high-confidence interactions, representing high-confidence physical contacts between the PET peaks. Specifically, the numbers of PET sequences that overlapped with PET peaks at both ends as well as the number of PETs within PET peaks at each end were counted. The PET count between two PET peaks represented the frequency of the interaction between the two genomic locations. A hypergeometric distribution was used to determine the probability of seeing at least the observed number of PETs linking the two PET peaks. A background distribution of interaction frequencies was then obtained by performing random shuffling of the links between two ends of PETs, and a cutoff threshold for calling significant interactions was set to the corresponding p value of the most significant proportion of shuffled interactions [false-discovery rate (FDR) (0.01 for Ronin and 0.05 for Pol2). The p values were corrected to control for multiple hypothesis testing by the Benjamini-Hochberg procedure. Two replicates were completed. The replicates were merged and then profiled, consistent with what has been done in publications and good ChIA-PET practice for minimizing false positives.^82^ Operationally, high-confidence interactions were defined as pairs of interacting sites with three independent PETs. The resolution of minimal interacting regions using ChIA-PET is limited by the width of the bound regions, which is determined by factors such as chromatin fragmentation, the binding pattern of the immunoprecipitated protein, and the peak-finding algorithms used. For Ronin, the average peak width is around 2 kb and the median is around 1.5 kb. For RNA Polymerase II, the average peak width is around 5 kb and the median is around 2.5 kb.

#### Arc plots

The arc plots used to illustrate chromosomal interactions along entire chromosomes were plotted with Protovis.^104^ Each dot represented an anchor region, and all pairs of interacting anchors were connected through arcs. The dots were positioned in order, but distances between the anchor regions in chromosomes were not drawn to scale. The size and color of a dot represented the total number of PETs in that anchor region. For each ChIA-PET pair, the number of PETs was counted for both anchor regions. The regional arc plots (zoomed into specific regions) were retrieved from the WashU Epigenome browser^106^ and only include interactions that start and end in the respective regions.

#### Mapping, alignment and classification of high-throughput sequencing data

High-throughput reads were aligned to the mouse genome (NCBI build 37, University of California at Santa Cruz build mm9) and visualized with the UCSC genome browser.^33^ Genes were annotated according to the RefSeq gene annotation (Mus musculus Annotation release 107). Ronin-bound genes were defined as genes whose promoter (TSS +/− 1 kb) has at least one Ronin peak. The list of human housekeeping genes was obtained by combining the list of housekeeping genes identified by Eisenberg and Levanon (2013)^41^ and by the Human Protein Atlas (https://www.proteinatlas.org/humanproteome/tissue/housekeeping). Mouse homolog genes of human housekeeping genes were obtained using Ensemble BioMart^97^ and considered “ Housekeeping genes”. Genes that belong to the Panther GO category GO:0008152 were considered “Metabolic genes”. Because Housekeeping genes only included protein-coding genes, only protein-coding genes were used to determine the overlap between Ronin-bound genes, housekeeping genes and metabolic genes for consistency. Protein-coding genes were identified with Ensemble BioMart. To determine statistical enrichment, hypergeometric testing was performed using dhyper function in R. To classify the interactions with respect to the overlap with promoter or enhancer elements, we defined promoters as the TSS +/− 2500 bp, and enhancers as the unions of the OCT4, SOX2, and NANOG ChIP-seq peaks that do not overlap with promoters.^25^ If a loop anchor overlapped both a promoter and an enhancer, the anchor was assigned to the promoter.

#### Ronin motif identification and distribution analysis

The Ronin binding motif was identified with MEME-ChIP 4.12.0 (http://meme.nbcr.net/meme/cgi-bin/meme-chip.cgi)^100^ by analyzing all ChIA-PET peaks.

#### Network and incidence plots

Network and incident plots were created in Mathematica (Wolfram Research). The edge list (newronin) for all Ronin interactions was converted it to a flat Mathematica list with the following command: edgesronin = Map[#[[1]] -> #[[2]] &, newronin] and the plots were generated with the commands Graph[edgesronin] and MatrixPlot[Incidence Matrix[edgesronin], PlotTheme ->"Classic"].

#### Visualization of Ronin signal intensities at genes (gene plot)

The Ronin signal intensities (Figure S3B) were analyzed from 2000 bp upstream of the transcription start site (TSS) to 2000 bp downstream of the transcription end site (TES) of a gene. The regions before the TSS and after the TES were evenly partitioned into 100 bins of 20 bp per bin. A fixed number of bins (300) was used for the entire gene from TSS to TES, regardless of the length of the gene. The averaged signal intensity in each bin of each individual gene was calculated first, and the averaged signal intensity for a group of genes was subsequently calculated based on the individual gene data (500 bins per gene).

#### Correlation plots and coefficients

For the correlation plots shown in Figure 2A, each chromosome was evenly partitioned into bins of 18.5 million base pairs in length. The number of Ronin-binding motifs, the sum of peak and the loop intensities (number of PETs in each bin) were calculated as the densities. The densities of all chromosomes were then concatenated in a random order to form the density distributions along the whole mm9 genome. The correlation coefficients, that are independent of the order of concatenation, were calculated.

#### Circularization assay

Circularization assays were performed as follows^17^: The substrate was generated by linearizing plasmid pTZ2073 (pGL3-PMax-Luc-PFntb; see “plasmid construction”) with NheI (NEB, R0131S) and XbaI (NEB, R0145S), which yielded a 5047-bp fragment with compatible ends and RBM-containing promoters on both ends, or pTZ1829 (pGL3-Enhancer, Promega) with SalI (NEB, R2138S) to generate a 5064 bp linear DNA substrate, which does not contain any Ronin-binding motif as control. The fragments were purified with a QIAquick gel extraction kit (Qiagen, 28704) according to the manufacturer’s protocol. Substrate (100 ng) was mixed with 1.358 μM of BSA (Sigma, A2153), recombinant Ronin (Prospec, PRO-2009), Ronin Thap domain (H00057215-Q01), Zfp143 (Abnova, H00007702-P01) or YY1 (Prospec, pro-2108 Prospec) in 1x T4 Ligase buffer in a final volume of 10 μL and incubated for 25 min at room temperature. T4 DNA ligase (200 units; NEB, M0202S) was added and the mixture was incubated for 20 min at room temperature. SDS containing loading dye was added to the samples to release the proteins and enzyme from the DNA. The samples were then separated by 0.5% agarose gel electrophoresis in TAE under native conditions and stained with ethidium bromide. The band intensities of the linearized substrate (5047 bp) and the monomeric circularized ligation product (which runs around the same level as the 3-kb marker) were quantified with the ImageJ software.^110^ The identity of the circular pGL2-PMax-Luc-PFntb monomer was confirmed by a NotI (NEB, R0189S) digest, which yielded one fragment (5047 bp) after linearization versus the two fragments (1280 bp and 3767 bp) that would be produced if the linear substrate were cut. The level of the circularized product was graphed relative to that found in control reactions run without the recombinant protein.

For the competition experiment, double-stranded competitors C1 (RBM sequence surrounded by a random sequence) or C2 (unspecific competitor with both RBM half-sites mutated)^50^ were generated by mixing and annealing equal amounts of 100 μM solutions (in 10 mM Tris pH 7.5, 1 mM EDTA, 50 mM NaCl) of Z10-605 (ACT AGC TCT CTG GGA ATT GTA GTT CGG CAA TCT CT) and Z10-606 (AGA GAT TGC CGA ACT ACA ATT CCC AGA GAG CTA GT) for competitor 1 (C1), or Z10-607 (ACT AGC TCT TGA TTC CGG AGC TGG AGG CAA TCT CT) and Z10-608 (AGA GAT TGC CTC CAG CTC CGG AAT CAA GAG CTA GT) for competitor 2 (C2). 0.25 μL of the annealed competitor oligonucleotides were used.

#### Atomic force microscopy

To visualize the DNA or protein-containing DNA loops by atomic force microscopy, we followed a modified protocol for the P element transposase^98^: The DNA substrate (a 1.7 kb DNA fragment containing the *Max* promoter with 3 RBMs and the *Fntb* promoter with 2 RBMs elements in antisense orientation, see Figure 5B), was generated by restriction of pTZ3023 (pSMaxFntbAS) with BglII (NEB, R0144S) and ApaI (NEB, R0114S). The 1.7 kb fragment was purified after gel electrophoresis with the Qiagen Gel Extraction kit following the manufacturer’s protocol. 100 ng of linearized plasmid alone or in combination with 400 ng of recombinant Ronin protein (Abcam, ab169918) were incubated in a final volume of 10 μL in the presence of 1x binding buffer, 350 mM KCl, 1 mM EDTA, 6% Glycerol, (all components of the Light Shift Chemiluminescent EMSA Kit, Thermo Fisher, 20148X). The samples were incubated for 10 min on ice, then 30 μL of binding buffer II (0.4 x T4 ligase buffer [NEB, B0202S], 20% Glycerol, 1 mM EDTA, and 2 mM GTP in H_2_O) were added and the samples were incubated for 30 min at room temperature. The samples were then diluted by adding 120 μL of 1x T4 Ligase buffer (NEB, B0202S) and 100 μL of the diluted samples were then dropped onto MICAs, that were pretreated by a procedure modified from Efremov et al.^111^ to preserve the native conformation of the protein-DNA samples. Briefly, the freshly cleaved MICAs were first treated with 100 μL of 0.1% (3-aminopropyl)ethoxysilane (APTES) solution for 15 min, rinsed three time with 1 mL of deionized water and dried with nitrogen gas. They were then treated with 1% glutaraldehyde for 15 min and washed and dried as in the previous step. The DNA or DNA-protein complexed were incubated on the Micas for 10 min, then washed with 1 mL of deionized water three times and dried with nitrogen gas. Imaging was done with an Atomic Force microscope (Bruker) at the Advanced Science Research Center at the City University of New York using tapping mode.

#### Coarse-grained molecular dynamics for ChIA-PET simulation

To corroborate the importance of the Ronin-binding motif in looping, coarse-grained molecular dynamics simulations of chromosomes were carried out with MATLAB.^102^ Each chromosome was binned into a set of nodes that could contain one or many RBMs. The Lennard-Jones potential was employed to simulate the interactions between nodes. For each pair of nodes, the force field was constructed as V_LJ_ = Σ [(r_m_/r)^12^-2(r_m_/r)^6^], where the potential well depth Σ is proportional to the sum of the number of RBM sequence elements of each pair of nodes and r_m_ is determined as follows: r_m_ ∝ r_chr_ [(e −(r_chr_/r_D_)+(1/r_chr_)], where r_chr_ is the linear base pair distance between the two nodes and r_D_ is the dephasing distance used to characterize the “stiffness” of the chromosome. In other words, the correlation between the movements of the two nodes will be almost lost and the motions of the two nodes will usually no longer be “in phase” if their distance reaches r_D_. Once the force field was built, the molecular dynamics simulations were performed at a proper temperature, T, in a canonical (NVT) ensemble. The velocity Verlet algorithm was used to integrate Newton’s equations of motion and the temperature was controlled using an Anderson thermostat.^112^ The results are shown as heatmaps, which qualitatively represent the experimental ChIA-PET results. To confine the nodes in the simulation box, a virtual flat repulsive boundary^113^ was introduced for all six directions: V_B_ = Σ(r_m_/r_B_), where r_B_ is the distance between a node and the boundary, and B could be ±x, ±y or ± z. The box length was chosen to be five times as the length of the simulated DNA, in order to minimize the spurious long-range interactions caused by the boundary effects. For each set of parameters, the simulation was carried out for 10,000 steps after equilibrium was reached. The fluctuation of the total energy was monitored during the process to ensure equilibrium of the molecular dynamics trajectory. A large number of parameter combinations was tested, and the optimal parameters were found by comparing the simulated PET pair length distributions to the experimental data. A normalized histogram of pair lengths was generated. The dephasing distance, r_D_, could theoretically be measured in experiments; to our knowledge, however, no experimental data are available. Moreover, the values of parameters such as temperature and timestep cannot be directly related to actual physiological conditions because of the arbitrary choice in the unit of Σ.

#### Venn diagrams

Venn diagrams were created with the BioVenn online application (http://www.biovenn.nl/index.php).^92^

#### Western blotting for ronin and tubulin

Cells were collected by trypsinization and centrifugation, washed once with DPBS and then lysed in 80 μL RIPA buffer (Thermo Fisher, 89900) supplemented with a protease inhibitor cocktail (Roche, 11873580001). Samples were incubated for 20 min on ice and cellular debris was removed by centrifugation at 20,000 g for 15 min at 4°C. Protein concentrations of supernatants were measured with a Pierce BCA kit (Thermo Fisher, 23225) according to the manufacturer’s instructions. Aliquots corresponding to 40 μg protein were mixed with Laemmli buffer, boiled for 5 min and separated on 10% polyacrylamide gels. The separated proteins were transferred to a PVDF membrane for 1 h at 100 V, blocked with 5% milk powder in DPBST for 45 min at room temperature and incubated overnight with a mixture of anti-Ronin (BD Biosciences, 562548, 1:1000) and anti-Tubulin (Sigma, T9026, 1:5000) or anti-LaminB1 (Proteintech, 66095, 1:5000) antibodies. The membrane was washed three times with DPBST and incubated with horseradish peroxidase-conjugated anti-mouse IgG (Promega, W4021, 1:2500). The membranes were washed as before, the bound antibody was detected with ECL (Amersham, RPN2109) and visualized with a Chemidoc XRS+ (Bio-Rad).

#### Mapping of chromatin interaction by chromosome conformation capture and PCR (3C-PCR)

Chromatin interactions were mapped by chromosome conformation capture followed by PCR (3C-PCR) according to a modified protocol.^114^ Cells (5 million per reaction) were resuspended in 5 mL of 10% FBS in DPBS, mixed with 5 mL of 3.7% formaldehyde in 10% FBS/DPBS, and incubated for 10 min at room temperature. The reaction was quenched by adding 500 μL of 2.5 M glycine (0.125 M final concentration). The cells were washed twice with ice-cold DPBS, flash frozen in liquid nitrogen and stored at −80°C. Cell pellets were thawed on ice for 15 min, lysed in 250 μL lysis buffer (10 mM Tris HCl, pH 8.0, 10 mM NaCl, 0.2% IGEPAL CA-630 (Sigma, I3021) and protease inhibitor (Roche Diagnostics, 1836170-001)) on ice for 30 min and nuclei were pelleted by centrifugation at 2500 g for 5 min at 4°C. Nuclei were washed once with 500 μL lysis buffer, resuspended in 50 μL 0.5% SDS, and incubated at 62°C for 7 min. The reactions were quenched by the addition of 170 μL of 1.7% Triton X-100 (Sigma, T8787) in H_2_O and incubated for 15 min at 37°C. 25 μL of CutSmart buffer (25 μL; 10x, NEB, B7204S) and 2 μL of MspI (100 U/μl; NEB, R0106M) were added. After 4 and 8 h of incubation at 37°C, additional 2 μL of MspI were added for a total of 600 U MspI. After an additional 12 h of incubation, the restriction was stopped by heat inactivation at 65°C for 30 min. Nuclei were collected as described above and resuspended in 1.2 mL T4 DNA ligase buffer supplemented with 0.83% Triton X-100 and 0.1 mg/mL BSA. T4 DNA ligase (2.5 μL, 2000 U/μl; NEB, M0202M) was added and samples were incubated for 6 h at room temperature. Nuclei were collected and resuspended in 300 μL 10 mM Tris-HCl, pH 8.0, 35 μL 5M NaCl, 35 μL 10% SDS and 50 μL proteinase K (20 mg/mL) and incubated for 30 min at 55°C. Samples were transferred to 62°C and incubated for 16 h to reverse the crosslinks, and the DNA was recovered by phenol-chloroform extraction and precipitation using 3M sodium acetate in 100% ethanol. The DNA was washed twice with 1 mL 70% ethanol, air dried and dissolved in 500 μL of 10 mM Tris-HCl, pH 7.5. 2 μL of the resuspended 3C library was PCR amplified with 0.5 μM of each primer and GoTaq Green Master Mix (Promega, M7123) in a total volume of 20 μL. The following primers were used for PCR, with the expected product sizes indicated: Max-Fntb PCR (250 bp), Z19-202 (Max 3C Left), CTC TCT CTC ACT CGC CCA TC and Z19-205 (Fntb 3C Right), TGG GTA GGA GAG GGT TGT TG; Mmachc-Toe PCR (132 bp), Z19-206 (Mmachc 3C Left), CGT ATT TCC GCC CTC TAT TG and Z19-211 (Toe1 3C Right), AAC TGC AAA ACC CGA ACT GT; Mmachc-Gpbp1l1 PCR (187 bp), Z19-206 (Mmachc 3C Left), CGT ATT TCC GCC CTC TAT TG and Z19-220 (Gpbp1Li 3C 2), TGT CGT GCA ATA GCC AAG AG; Gpbp1l1-Toe (139 bp), Z19-221(Gpbp1Li 3C 2), AAC TGA GGG AGG GAA GCA TT and Z19-211 (Toe1 3C Right), AAC TGC AAA ACC CGA ACT GT; Myc-Pvt1 PCR (249 bp), Z19-240 (Myc 3C left), TTT TTC CTC CTC TCG CTT CC and Z19-242 (Pvt13C left), AAA GCC TCT GCA AAG AAC GA; Ubtf-Slc25a29 PCR (154 bp), Z19-229 (Ubtf 3C left), ATC TCG GGC TTT GTC TGG AG and Z19-231 (Slc25a39 3C left), GCT GTC CCA CAC TCT GAT GA; and Phc-Promoter-Enhancer PCR (189 bp), Z19-217 [Phc1 15 3C (Promoter)], TGT GCC CGA AGC GAG CGG ACT TGG TAA G and Z19-227 [Phc1 48 3C (Enhancer)], CAT CTA CCT ATG TAG TCG AGG CAA CCA AGC.^89^ The PCR conditions were as follows: 95°C for 5 min, followed by 40 cycles of 1 min of denaturation at 95°C, 45 s of annealing at 60°C and 2 min of extension at 72°C and a final extension for 6 min at 72°C. The PCR products (20 μL of each reaction) were resolved on 2% TAE agarose gels and stained with ethidium bromide. The band intensities were quantified with the ImageJ software.

#### Chromatin immunoprecipitation and qPCR

Cells (5x10^6^) were washed with DPBS, resuspended in 5 mL 10% FBS in DPBS, mixed with 5 mL of 4% formaldehyde and fixed for 10 min with rotation at room temperature. The reaction was quenched with 0.125 M glycine, the cells were washed twice with ice-cold DPBS and chromatin immunoprecipitation was performed^115^: Briefly, cells were lysed in 1 mL of 1% SDS, 50 μM Tris-HCl, pH 8.0, 10 μM EDTA, and protease inhibitors (Roche Diagnostics, 1836170-001). The lysates were sonicated to obtain chromatin fragments of <1 kb, and debris was removed by centrifugation at 5,000 rpm for 10 min at 4°C. A 10th of each lysate was saved as the input sample and stored at −80°C. The remainder of each sample was precleared with 30 μL protein G-Sepharose for 30 min at 4°C. The samples were split (450 μL each) and used for IgG control IP and Ronin-IP. Each portion was mixed with 550 μL ChIP dilution buffer (0.01% SDS, 1.1% Triton X-100, 1.2 mM EDTA, 16.7 mM Tris-HCl, pH 8.0, 150 mM NaCl) to reach a final volume of 1 mL. Either 2.5 μg of mouse Ronin (562548, BD Biosciences) or mouse IgG (SC-2025, Santa Cruz) were added, the samples were incubated with 30 μL protein G-Sepharose for 2 h at 4°C and immune complexes were collected by centrifugation at 1,000 rpm for 1 min at 4°C. The samples were washed with 1 mL each of various buffers, as follows: twice with ChIP dilution buffer; once with 0.1% SDS, 1% Triton X-100, 2 mM EDTA, 20 mM Tris-HCl, pH 8.0, and 150 mM NaCl; once with of 0.1% SDS, 1% Triton X-100, 2 mM EDTA, 20 mM Tris-HCl, pH 8.0, and 500 μM NaCl; once with 0.25 M LiCl, 1% NP-40, 1% Na-deoxycholate, 1 mM EDTA and 10 mM Tris-HCl, pH 8.0; and twice with 10 mM Tris-HCl, pH 8.0, and 1 mM EDTA. Each wash was performed for 3 min with rotation at 4°C, followed by centrifugation for 1 min. After the last wash, the chromatin was eluted from the beads with 500 μL of ChIP elution buffer (1% SDS, 0.1 M Na_2_CO_3_). Samples were vortexed and incubated for 10 min, and the beads were removed by centrifugation for 3 min at 10,000 rpm. ChIP elution buffer (400 μL; 1% SDS, 0.1 M NaHCO_3_) was added to each input sample, and the input and ChIP samples were subjected to crosslink reversal by the addition of 20 μL of 5 M NaCl and incubation overnight at 65°C. Following the addition of 10 μL 1 M Tris-HCl, pH 6.5, 10 μL 0.5 M EDTA, and 10 μg proteinase K, the samples were incubated at 48°C for 1 h. The DNA was recovered by phenol-chloroform extraction followed by ethanol precipitation. The precipitate was dissolved in 100 μL TE with 10 μg/mL RNase and incubated at 37°C for 30 min. Triplicate aliquots (1 μL each) were amplified by PCR using SybrGreen PCR Master Mix (Applied Biosystems) and 0.5 μM of each oligo in a final volume of 10 μL. Reactions were run for 40 cycles using a CFX384 real-time machine (Biorad). qPCR was performed in triplicate and data obtained from the ChIP samples were normalized to those obtained from the input DNA. The oligos were as follows: Snx14 RBM (wt: 186 bp): Z10-746 (Snx14-F), CCC ACT TTC CTT ATC CGG AAG TCC and Z10-747 (Snx14-R), TTT CCT CCT CTC CCT TAG GTG TGG; F08Rik RBM (wt: 214 bp): Z10-742 (F08Rik-F), AAG ATC CTA ACT GCA CCA CCA AGG and Z10-743 (F08Rik-R), TAG TGG CCA AGT ATA CAA CAT CGG; and Ubxn RBM (wt: 227 bp): Z10-738 (Ubxn4-F), ACA GCG TTG ATG TGC TCA CTA TGC and Z10-739 (Ubxn4-R), TCG GCT AGG TAA GTC CGT AAG TGC.

#### RNA-seq analyses

2x10^6^ cells were collected for each condition. RNA isolation and RNA-seq analyses were performed by Active Motif. Briefly, 42-nt sequence reads were generated using Illumina NextSeq 500 and mapped to the mouse genome the genome using the STAR algorithm with default settings. After obtaining the gene table containing the fragment (or read) counts of genes, differential analysis to identify statistically significant differential genes was performed using DESeq2.^96^ After a differential test had been applied to each gene except the ones with zero counts, the p value of each gene was calculated, and multiple testing adjustment performed. Genes were considered differential genes, and therefore, Ronin-regulated, if the FDR (i.e., adjusted p value) was lower than 0.05. Heatmaps were generated using Excel, Seqminer^105^ and Prism v8.3.0. To report an accurate absolute value of the log_2_(fold change) in the heat-map, the read count of genes with low expression (TPM lower than 0.5) and genes whose FDR = N/A (mostly lowly expressed genes) were reported to be equal to 1. The Scatterplot in Figure 4B was produced using the plot function in R version 4.1.2^103^ and only genes with TPM higher than 0.5 were plotted. Gene ontology (GO) analyses were performed using Panther v17.0 using the Overrepresentation Test, GO-Slim Biological Process.^116,95^

#### Gene set enrichment analyses (GSEA)

GSEA was performed using the GSEAPreranked module of the GenePattern online tool^99^ with the recommended parameters (1000 permutations, weighted scoring scheme, Max_probe collapsing mode for probe sets with more than one match, meandiv normalization mode.

#### RNA fluorescence *in situ* hybridization (RNA FISH)

The RNA FISH procedure was performed according to the protocol by Biosearch Technology.^70^ Briefly, cells were grown on gelatinized coverslips, washed with Dulbecco’s Phosphate Buffered Saline (DPBS, 14190-144, Gibco), fixed with 3.7% formaldehyde and permeabilized with 70% ethanol, washed with Wash Buffer A (20% Stellaris RNA FISH wash buffer A (SMF-WA1-60) and 10% deionized formamide (Sigma, F9037) for 5 min, and incubated with 100 μL of a 1:100 dilution of the fluorescent probes (12.5 μM stock solution in TE buffer) in hybridization buffer (90% Stellaris RNA FISH hybridization buffer (SMF-HB1-10) and 10% deionized formamide) for 4 h at 37°C in a humid chamber. The cells were washed with wash buffer A for 30 min at 37°C in the dark; nuclei were stained with 5 ng/mL 4,6-diamidino-2-phenylindole (DAPI) in wash buffer A for 30 min in the dark; and the cells were washed with Wash Buffer B (Stellaris, SMF-WB1-20) for 5 min at room temperature and then mounted with Vectashield mounting medium (Vector laboratories, H-1000). Stellaris FISH probes (designed to target intronic regions whenever possible, Tables S1J and S1K) were labeled as follows: Quasar570: Ubxn4, 4930430F08Rik, Zfp949, Rabac1, Rhoc, Dusp27; Quasar670: Gsk3a (Figure 3) R3hdm1, Tmtc3, Snx14, Capza1, Pou2f1. TAMARA C9: Gsk3a (Figure 6) While we were able to use intron-specific probes for each probe in most cases that gave us very clear signals, we were limited in the probe-design and had to include exons in some cases (e.g., Gsk3a). In such instances, we carefully analyzed each cell and measured the closest pair of signals within the DAPI stained nucleus. The gene loci for the RNA FISH analyses were chosen because they are Ronin targets that are looped at high frequency but did not show drastic transcriptional changes and they were distant enough from each other based on the linear sequence of the genome, ensuring the proper study of interactions. Images were acquired with an Axioimager Z2M Plan Apochromat 63x/1.4 NA (Zeiss) and a digital black/white camera. Images were processed with the Zen 2.3 lite software (Zeiss) and the distances between each pair of differently labeled sites of active transcription observed for at least 50 events per condition were measured.

#### Immunofluorescence staining of ronin protein

Cells were fixed with 4% paraformaldehyde for 10 min at room temperature, permeabilized in 0.5% Triton X-100/DPBS buffer for 10 min at RT, washed three times in DPBS, blocked in 4% BSA/DPBS, and incubated with the mouse anti-Ronin (Becton Dickinson, BDB562548) antibody (1:500 in 4% BSA/DPBS) for 1 h at room temperature. The cells were washed three times with DBPS and then incubated with anti-mouse Alexa Fluor 488 (Invitrogen) as secondary antibodies (1:500 in 4% BSA/DPBS) for 1 h at room temperature. The cells were washed as before, stained with Hoechst and imaged using the LSM980 microscope with Ayriscan (Zeiss) at the Keck Microscopy core at the Whitehead Institute. All images were acquired at a pixel size of 15.3 nm. Images were processed using the FIJI (ImageJ) software.^110^

#### Immunofluorescence combined with RNA fluorescence *in situ* hybridization (FISH)

Cells were grown on Poly-L-ornithine and laminin-coated 24-well glass-bottom dishes, fixed with 4% Paraformaldehyde in DPBS for 10 min at room temperature, washed with DPBS, fixed with 4% Paraformaldehyde in DPBS for 10 min at room temperature, washed three times with DPBS and permeabilized with 0.5% Triton X-100 in RNase-free PBS at RT for 10 min. The cells were then washed with RNase-free PBS three times for 5 min, blocked with 4% BSA in RNase-free PBS for 10 min, incubated with primary antibodies anti-RNA Pol II CTD repeat phosphoS5 (Abcam, ab5131) and mouse anti-Ronin (Becton Dickinson, BDB562548) diluted 1:500 in RNase-free PBS overnight at 4°C. After three washes in RNase-free PBS for 5 min, cells were incubated with the secondary antibodies anti-mouse-AF647 (Invitrogen, A21235) and anti-rabbit-AF488 (Invitrogen, A11008), respectively, diluted 1:500 in RNase-free PBS for 1 h at RT and washed three times in RNase-free PBS for 5 min. Cells were then fixed again with 4% PFA in RNase-free PBS for 10 min at room temperature, washed three times with RNase-free PBS for 5 min. Then RNA FISH procedure was performed according to the protocol by Biosearch Technology as described above starting at the “Wash buffer A” step. Cells were imaged using the LSM980 microscope with Ayriscan (ZEISS) at the Keck Microscopy core at the Whitehead Institute. Images were processed using the FIJI (ImageJ) software.^110^ To quantify RNA Polymerase II signal at the RNA FISH spot, the RNA FISH spot in the nuclei was circled using the oval function and IF fluorescence intensity was measured with the Measure tool.

#### Recombinant protein purification

cDNA encoding the Ronin was fused to 5′ 6xHIS followed by mEGFP and a 14 amino acid linker sequence “GAPGSAGSAAGGSG.” Expression constructs were sequenced to ensure sequence identity. Plasmids containing the protein of interest were transformed into LOBSTR cells and proteins were purified^37^: A fresh bacterial colony was inoculated into LB medium containing kanamycin and chloramphenicol and grown overnight at 37°C. Cells were diluted 1:30 in 500mL LB with freshly added kanamycin and chloramphenicol and grown 2.5 h at 16°C. IPTG was added to reach a concentration of 1 mM and growth continued for 20 h. Pellets from 500 mL cells were resuspended in 15 mL of Buffer A (50 mM Tris pH7.4, 500 mM NaCl), complete protease inhibitors (Roche, 11873580001) and sonicated (ten cycles of 15 s on, 60 s off). The lysate was cleared by centrifugation at 12,000g for 30 min at 4°C and added to 1 mL of Ni-NTA agarose (Invitrogen, R901-15) pre-equilibrated with 10x volumes of Buffer A. Tubes containing the agarose lysate slurry were rotated at 4°C for 1.5 h. The slurry was centrifuged at 3,000 rpm for 10 min. The resin was washed twice with 5 mL of Buffer A followed by two washes with 5 mL Buffer A containing 50 mM imidazole. The protein was eluted with 3 × 2 mL Buffer A containing 250 mM imidazole incubating rotating for 10 or more minutes each cycle at 4°C. Each eluate was run on a 12% Bis-Tris acrylamide gel. Fractions containing protein of the correct size were dialyzed against two changes of buffer containing 50 mM Tris 7.4, 500 mM NaCl, 10% glycerol and 1 mM DTT at 4C. Any precipitate after dialysis was removed by centrifugation at 3.000rpm for 10 min.

#### Production of fluorescent DNA

The fluorescent DNA containing 20 Ronin-binding motifs was synthetized by IDT and cloned into the pUC19 vector using HiFi Assembly (NEB).^39^ Cy5-labeled M13(21) (/5Cy5/TGTAAAACGACGGCCAGT) and M13 reverse (/5Cy5/CAGGAAACAGCTATGAC) primers were used to amplify the synthetic DNA sequence by PCR, yielding a fluorescently labeled PCR product. The fluorescent PCR products were purified twice, first with the QIAGEN PCR purification kit and then with the NEB Monarch PCR purification kit.

#### *In vitro*-droplet assays

For all droplet assays, DNA was included at 50 nM, mEGFP-Ronin protein at 1250 nM, BFP-MED1-IDR^90^ protein at 150 nM and mCherry-CTD protein^91^ at 50 nM. No crowding agents were used. The final buffer conditions were 50 mM Tris-HCl pH 7.5, 150 mM NaCl, 10% glycerol, 1 mM DTT. The solution was mixed in PCR-strips and immediately transferred to 384 glass-bottom multi-wells. Following a 30-min incubation, samples were imaged with an Andor confocal microscope using a 100× objective. Images presented in Figures 6H and 6I are of droplets settled on the glass, while images presented in Figure 6J are droplets floating in solution.

#### Fluorescence recovery after photo bleaching (FRAP)

FRAP experiments were performed^37^: the Ronin-GFP expressing cells were imaged using the LSM980 microscope with Ayriscan detector (Zeiss) with 488nm laser. Bleaching was performed using the 488nm laser at 100% power and images were collected every 2.5 s. Fluorescence intensity was measured using FIJI. Values are reported relative to pre-bleaching time points.

### QUANTIFICATION AND STATISTICAL ANALYSIS

Statistical details of experiments are described in the figures, figure legends, and methods. Data analyses were performed with Excel (Microsoft) if not otherwise stated. T-tests were done with the t test (unpaired, two-tailed) function of Excel. The exact numbers for each experiment (n) are provided and defined within the corresponding figures or figure legends. Significance is indicated with *p < 0.05, **p < 0.01, ***p < 0.001 and ****p < 0.0001. Boxplots were generated with BoxPlotR (http://shiny.chemgrid.org/boxplotr/). Center lines show the medians. Box limits indicate the 25th and 75th percentiles, as determined by the R software. Whiskers extend to 5th and 95th percentiles, and outliers are represented by dots. Violin plots were generated using the vioplot package. White circles show the medians; box limits indicate the 25th and 75th percentiles as determined by R software; whiskers extend 1.5 times the interquartile range from the 25th and 75th percentiles; polygons represent density estimates of data and extend to extreme values.

## Supplementary Material

1

2

3

4

5

## Figures and Tables

**Table:** KEY RESOURCES TABLE

REAGENT or RESOURCE	SOURCE	IDENTIFIER
Antibodies		
Anti-Ronin (Clone P56-507)	Becton Dickinson	562548
Anti-RNA polymerase II CTD repeat YSPTSPS (Clone 8WG16)	Abcam	Ab817
Anti-RNA Pol II CTD repeat phosphoS5	Abcam	Ab5131
Anti-Tubulin	Sigma	T9026
Anti-Lmnb	Proteintech	66095
Anti-mouse IgG - horseradish peroxidase-conjugated	Promega	W4021
Mouse IgG	Santa Cruz	SC-2025
Goat anti-Rabbit IgG (H + L) Cross-Adsorbed Secondary Antibody, Alexa Fluor 488	Invitrogen	A11008
Goat anti-Mouse IgG (H + L) Cross-Adsorbed Secondary Antibody, Alexa Fluor 647	Invitrogen	A21235
Bacterial and virus strains		
LOBSTR-BL21(DE3)-RIL	Kerafast	EC1002
Top10	Invitrogen	C404010
Chemicals, peptides, and recombinant proteins		
2-mercaptoethanol	Sigma-Aldrich	63689
(3-aminopropyl)ethoxysilane (APTES)	Sigma-Aldrich	A3648
4-Hydrotamoxifen	Sigma-Aldrich	T176
Bovine serum albumin, BS	Sigma-Aldrich	A2153
BSA	Sigma-Aldrich	A2153
Buffer EB	Qiagen	19086
Cytofix/Cytoperm solution	Becton Dickinson	BDB554714
CutSmart buffer	New England Biolabs	B7204S
DAPI, 4,6-diamidino-2-phenylindole	Sigma-Aldrich	D9542
DMEM, High glucose	Gibco	1569010
DMSO	Sigma-Aldrich	D8418
DPBS	Gibco	14190144
DTT	Sigma-Aldrich	D9779
Dynabeads M-280 Streptavidin	Invitrogen	11205D
ECL Western blotting detection reagent	Amersham Biosciences	RPN2109
EDTA, pH 8.0	Sigma-Aldrich	AM9260G
Ethanol	Sigma-Aldrich	E7023
Ethylene glycol bis(succinimidyl succinate)	Thermo Fisher	21565
Exonuclease	New England Biolabs	M0262S
Formaldehyde	Sigma-Aldrich	F8775
Formamide, deionized	Sigma-Aldrich	F9037
Fetal bovine serum, FBS	Sigma-Aldrich	F4135
Gelatin	Sigma-Aldrich	G1890
Glacial acetic acid	Fisher Scientific	A38-212
GlutaMax Supplement	Gibco	35050061
Glutaraldehyde	Sigma-Aldrich	G6257
Glycine	Sigma-Aldrich	G8790
Glycerol	Sigma-Aldrich	G9012
GoTaq Green Polymerase	Promega	M7123
GTP, Guanosine 5′-triphosphate sodium salt hydrate	Sigma-Aldrich	G8877
HEPES-KOH pH 7.5	Sigma-Aldrich	15630080
Hoechst	Sigma-Aldrich	B2883
Hygromycin B	Invitrogen	10687–010
IGEPAL CA-630	Sigma-Aldrich	3021
Klenow Fragment (3’→5’ exo-)	NEB	M0212
Laemmli buffer	Bio-Rad	1610747
Lambda nuclease	NEB	M0293S
Leukemia inhibitory factor	Millipore	ESG1107
Lipofectamine 2000	Invitrogen	52887
Lithium chloride	Sigma-Aldrich	L9650
MEM Non-essential amino	Gibco	11140050
Methanol	Sigma-Aldrich	A412-4
NP-40	Sigma-Aldrich	74385
Paraformaldehyde	Sigma-Aldrich	158127
Penicillin-Streptomycin solution	Gibco	15140122
Phenol:chloroform:isoamyl alcohol	Sigma-Aldrich	77617
PMSF, Phenylmethylsulfonyl fluoride	Sigma-Aldrich	P7626
Potassium hydroxide solution	Sigma-Aldrich	P4494
Prolong Dimond antifade mounting medium	Invitrogen	P36965
Protease inhibitor, Complete Mini, EDTA-free	Roche Diagnostics	1836170–001
Proteinase K	Invitrogen	AM2548
Protein G Dynabeads	Life Technologies	10004D
Protein G-Sepharose	GE Healthcare	17-0618-01
Recombinant Ronin protein	Abcam	ab169918
Recombinant Ronin protein	Prospec	PRO-2009
Recombinant Ronin Thap domain	Abnova	H00057215-Q01
Recombinant YY1 protein	Prospec	PRO-2108
Recombinant Zfp143	Abnova	H00007702-P01
Restriction endonuclease SalI	New England Biolabs	R3138S
Restriction endonuclease XbaI	New England Biolabs	R0145S
Restriction endonuclease NheI	New England Biolabs	R0131L
Restriction endonuclease NotI	New England Biolabs	NotI (NEB, R0189S)
Restriction endonuclease BglII	New England Biolabs	NEB, R0144S)
Restriction endonuclease ApaI	New England Biolabs	R0114S
Restriction endonuclease MspI	New England Biolabs	R0106M
RIPA buffer	Thermo Fisher	89900
RNase A	Sigma-Aldrich	R4642
RNA FISH wash buffer A	Stellaris	SMF-WA1-60
RNA FISH Wash Buffer B	Stellaris	SMF-WB1-20
RNA FISH hybridization buffer	Stellaris	SMF-HB1-10
SDS, 10%	Gibco	15553–027
Sodium acetate, 3M	Sigma-Aldrich	S7899
Sodium chloride	Sigma-Aldrich	S5150
Sodium deoxycholate	Sigma-Aldrich	D6750
Sodium carbonate, Na_2_CO_3_	Sigma-Aldrich	31432-250G-R
SybrGreen PCR Master Mix	Applied Biosystems	4309155
TAE	Bio-Rad	1610743
T4 DNA polymerase	New England Biolabs	M0203
T4 Ligase	New England Biolabs	M0202 S/M
T4 ligase buffer	New England Biolabs	B0202S
Tris-HCl, pH 7.5	Invitrogen	15567–027
Tris-HCl pH 8.0	Invitrogen	15568–025
Triton X-100	Sigma-Aldrich	T8787
Trypsin-EDTA 0.05%	Gibco	25300120
Vectashield mounting medium	Vector laboratories	H-1000
Critical commercial assays		
RNeasy Mini Kit	Qiagen	74104
QIAquick Gel Extraction Kit	Qiagen	28704
DNeasy Blood and Tissue kit	Qiagen	69506
QIAquick PCR purification kit	Qiagen	28104
QIAprep Spin Miniprep Kit	Qiagen	27106
Plasmid Maxi kit	Qiagen	12163
Nextera DNA Library preparation kit	Illumina	FC-121-1030
Zymo DNA purification columns	Zymo Research	D4003
AMPure beads	Beckman Coulter	A63880
Pierce BCA kit	Thermo Fisher	23225
Light Shift Chemiluminescent EMSA kit,	Thermo Fisher	20148X
Deposited data		
Ronin ChIA-PET mouse ES cells	This study, GEO	GSE136145; GSM4041606
Pol2 ChIA-PET Control cells	This study, GEO	GSE136145; GSM4041604
Pol2 ChIA-PET *Ronin*-knockout cells	This study, GEO	GSE136145; GSM4041605
Mouse genome NCBI build 37 (University of California at Santa Cruz build mm9)	UCSC genome browser^33^	http://genome.ucsc.edu/
Mus musculus Annotation release 107	NCBI	N/A
Housekeeping genes	Eisenberg and Levanon^41^	N/A
Housekeeping genes	Human Protein Atlas	https://www.proteinatlas.org/humanproteome/tissue/housekeeping
Metabolic genes	PANTHER	GO:0008152
Hi-C promoter-capture data	Schoenfelder et al.^68^	https://www.ebi.ac.uk/biostudies/arrayexpress/studies/E-MTAB-6585?query=E-MTAB-6585
Ronin target genes	Dejosez et al.^50^	N/A
Ronin target genes	Hnisz et al.^21^	N/A
Experimental models: Cell lines		
R1 mouse ES cells	ATCC	SCRC-1011
CreERT2; Ronin^flox/flox^	This study	ZCL1032
Ronin^flox/flox^	This study	ZCL1040
D3-Ronin-GFP (ZCL1029)	Dejosez et al.^61^	ZCL1029
DR4 fibroblasts	GlobalStem	GSC-6204G
Irradiated CF1 mouse fibroblasts	GlobalStem	GSC-6001G
Experimental models: Organisms/strains		
ROSA26^Cre–ERT2/+^	Guo et al.^78^	N/A
Ronin^loxP/+^	Dejosez et al.^61^	N/A
Ronin^+/−^	Dejosez et al.^61^	N/A
Oligonucleotides		
Oligonucleotides designed for this study	This study, IDT	Table S4
Bridge Linker-F/5Phos/CGC GAT ATC/iBiodT/TAT CTG ACT	Hnisz et al.^21^	N/A
Bridge Linker-R/5Phos/GTC AGA TAA GAT ATC GCG T	Hnisz et al.^21^	N/A
Nextera primers	Illumina	FC-121-1030
M13R GTAAAACGACGGCCAGT	Macrogen	M13R
M13R-pUC CAGGAAACAGCTATGAC	Macrogen	M13R-pUC
Phc1 15 3C (Promoter), TGTGCCCGAAGCGAGCGGACTTGGTAAG	Kagey et al.,^89^ IDT	Z19-217
Phc1 48 3C (Enhancer) CATCTACCTATGTAGTCGAGGCAACCAAGC	Kagey et al.,^89^ IDT	Z19-227
Recombinant DNA		
pGL3-Basic-MaxPro	Dejosez et al.^50^	pTZ1841
pGL3-PMax-Luc-PFntb	This study	pTZ2073
pSMaxFntbAs	This study	pTZ3023
pCas9-gSnx14	This study	pTZ3019
pCas9-gRikF08	This study	pTZ3020
pCas9-gUbxn4	This study	pTZ3021
pGL3-Enhancer	Promega	E1771
pET6H-Ronin-GFP	This study	pTZ3028
pBFP-MED1-IDR	Klein et al.^90^	N/A
pET6H-mC-CTD (mCherry-CTD)	Guo et al.^91^	pTZ3027
pRBM20	This study	N/A
Software and algorithms		
BioVenn	Hulsen et al.^92^	http://www.biovenn.nl/index.php
Bowtie version 1.1.1	Langmead et al.^93^	https://bioweb.pasteur.fr/packages/pack@bowtie@1.1.1
Cutadapt	Krueger and Andrews^94^	https://github.com/marcelm/cutadapt
PANTHER version 17.0 Released 2022-02-22	Thomas et al.^95^	http://www.pantherdb.org
DESeq2	Love et al.^96^	https://bioconductor.org/packages/release/bioc/html/DESeq2.html
Ensemble BioMart	Kinsella et al.^97^	https://genome.ucsc.edu/
ImageJ software	Schindelin et al.^98^	N/A
GenePattern GSEA online tool	Reich et al.^99^	https://www.genepattern.org
MEME-ChIP 4.12.0	Machanick et al.^100^	https://meme-suite.org/meme/
MACS 1.4.2	Zhang et al.^101^	https://bioweb.pasteur.fr/packages/pack@macs@1.4.2
Mathematica	Wolfram Research	https://www.wolfram.com/mathematica/
MATLAB	MATLAB^102^	https://www.mathworks.com/products/matlab.html
R version 4.1.2	R Core Team^103^	https://www.r-project.org
Protovis	Bostock and Heer^104^	https://github.com/mbostock/protovis
seqMINER	Ye et al.^105^	https://sourceforge.net/projects/seqminer/
UCSC genome browser	Kent et al., 2002^33^	https://genome.ucsc.edu/
WashU Epigenome browser	Zhou et al., 2012^106^	https://epigenomegateway.wustl.edu/
Other		
RNA-seq service	Active Motif	N/A
100x100 paired-end sequencing service (Illumina HiSeq 2500)	Genome Tech Core at the Whitehead Institute for Biomedical Research	N/A

## Highlights

- Housekeeping genes lack distal enhancer interactions
- Ronin assembles housekeeping gene promoters
- Ronin promoter assemblies are analogous to enhancer function
- Ronin assemblies explain why housekeeping genes lack enhancers

## INCLUSION AND DIVERSITY

We support inclusive, diverse, and equitable conduct of research.
