# Supplementary material for: Regulatory architecture of housekeeping genes is driven by promoter assemblies
Source: Cell Rep. Author manuscript; Available in PMC 2023 Jul 9. (PMC10329844; doi:10.1016/j.celrep.2023.112505)
Supplement: 1 [file NIHMS1905380-supplement-1.pdf]

Cell Reports, Volume *42*

Supplemental information

Regulatory architecture of housekeeping genes

is driven by promoter assemblies

Marion Dejosez, Alessandra Dall'Agnese, Mahesh Ramamoorthy, Jesse Platt, Xing Yin, Megan Hogan, Ran Brosh, Abraham S. Weintraub, Denes Hnisz, Brian J. Abraham, Richard A. Young, and Thomas P. Zwaka

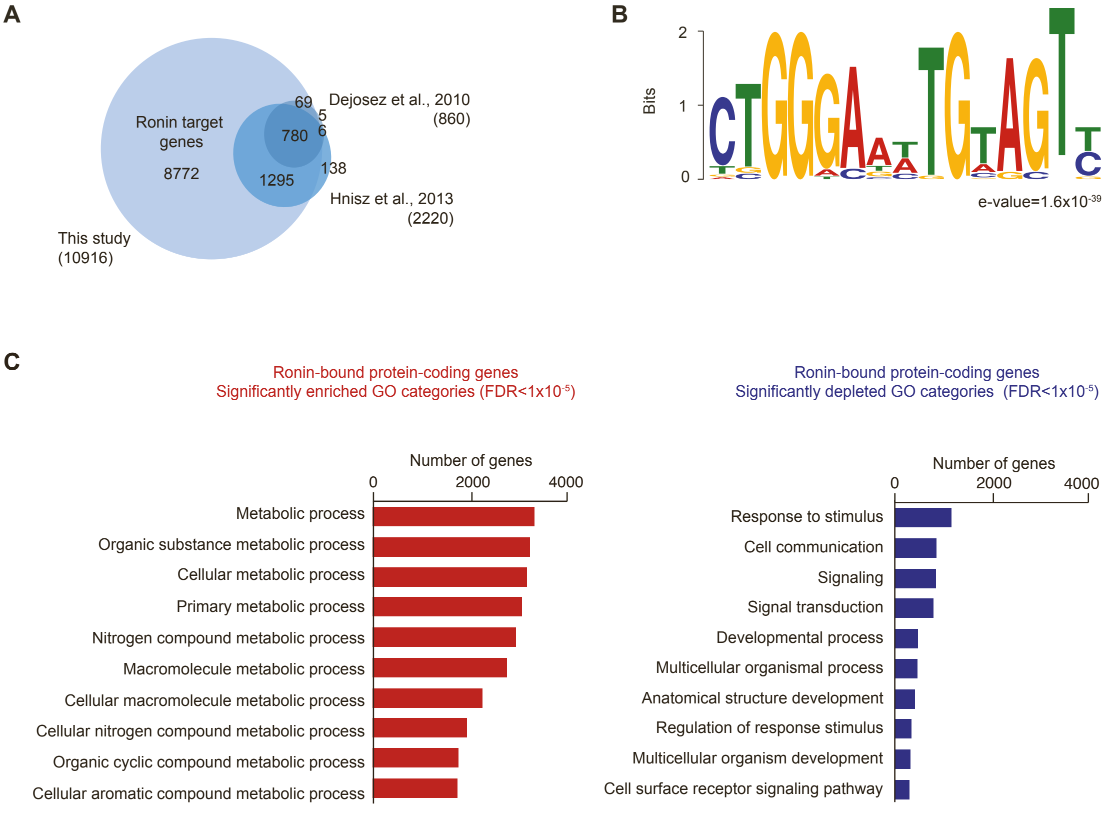

Figure S1. Ronin-binding characteristics and motif. Related to Figure 1 and Table S1.

(A) Venn diagram showing overlap of the newly identified Ronin-bound protein-coding genes with previously published Ronin target gene sets.
(B) Ronin DNA-binding motif within newly identified Ronin-bound peaks.
(C) Gene ontology analysis of Ronin-bound genes. Shown are the top 10 categories with the highest gene counts that were significantly enriched or depleted among protein-coding Ronin-target genes.

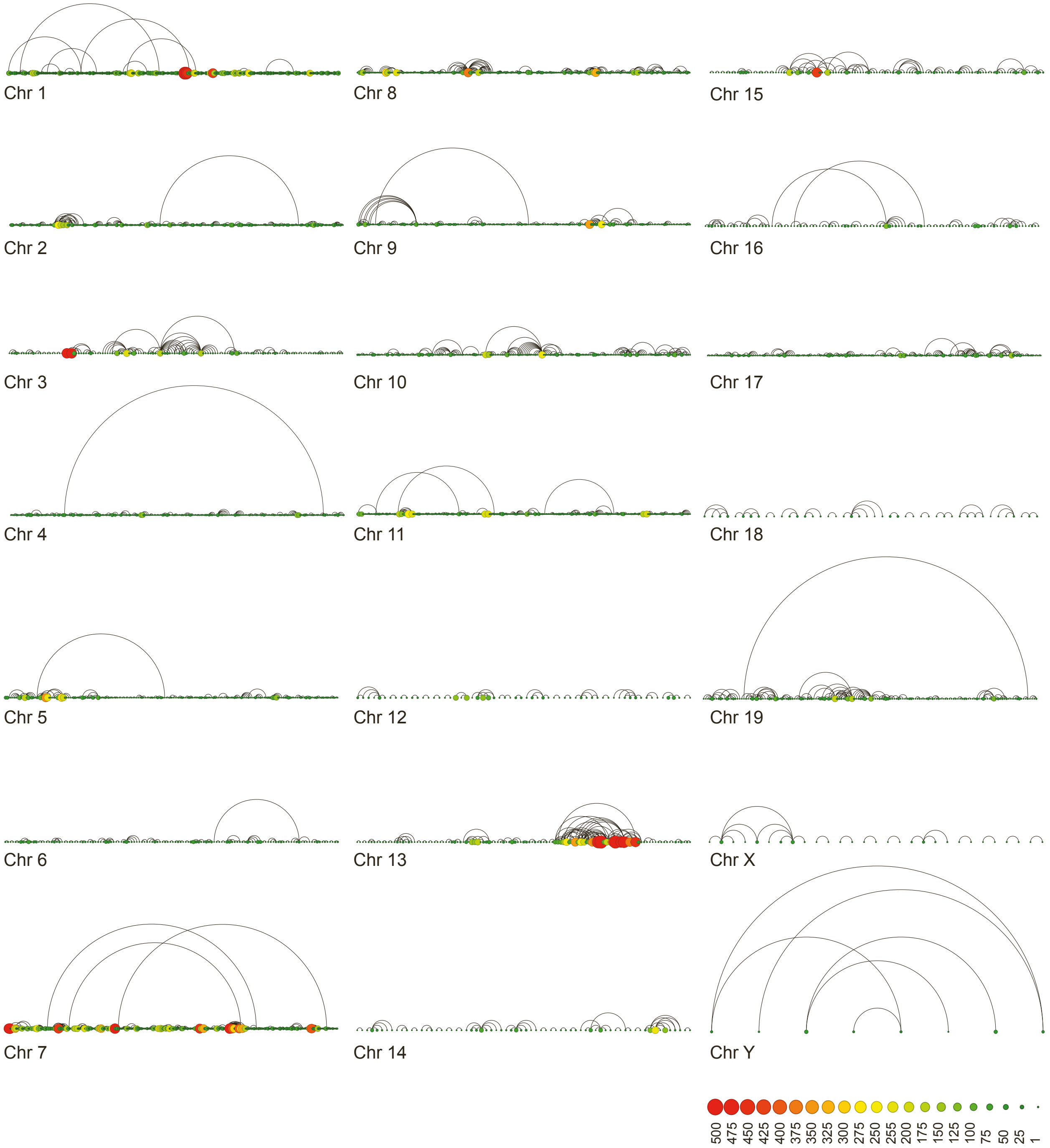

Figure S2. Arc plots of chromosomal interactions identified by Ronin ChIA-PET. Related to Figure 1 and Table S1.

Each dot represents an anchor region, and all pairs of interacting anchors are connected through arcs. The color and size of each dot correlate with the cumulative PET count for each anchor. Note that the distances between the anchor regions within individual chromosomes are not drawn to scale. Chr, chromosome.

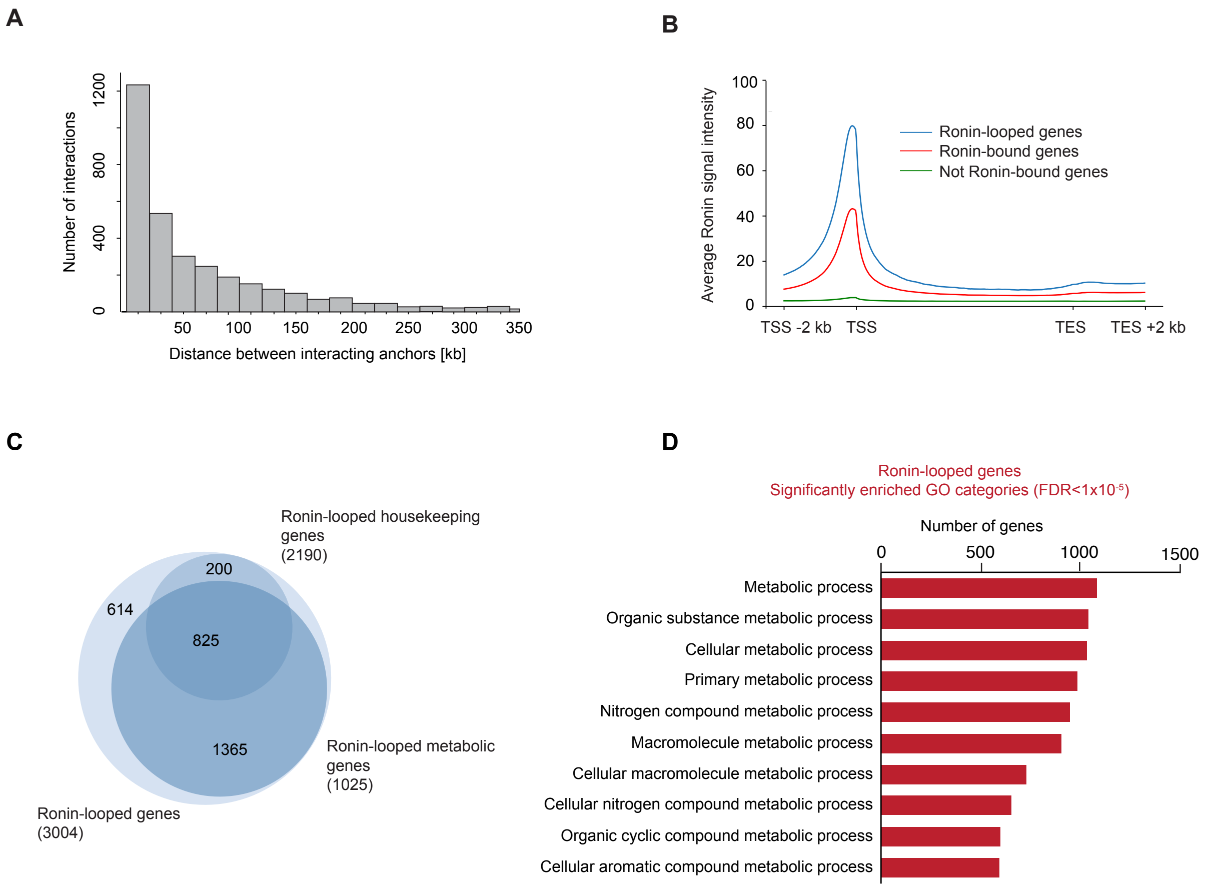

Figure S3. Ronin is associated with chromosomal sites that interact *in vivo*. Related to Figure 1 and Table S1.

(A) Histogram of distances between interacting Ronin anchors.
(B) Average Ronin peak intensities of indicated gene sets from 2 kb upstream of transcription start sites (TTS) to 2 kb downstream of transcription end sites (TES).
(C) Venn diagram showing the overlap of Ronin-looped genes with Ronin-looped housekeeping ($p=1.5^{149}$) and metabolic genes ($p=5.1^{164}$).
(D) Gene ontology for Ronin-looped genes. Shown are the top 10 categories with the highest gene counts that were significantly enriched among protein-coding Ronin-looped genes.

kb, kilobase pairs; P, promoter; TSS, transcription start site; TES, transcription end site.

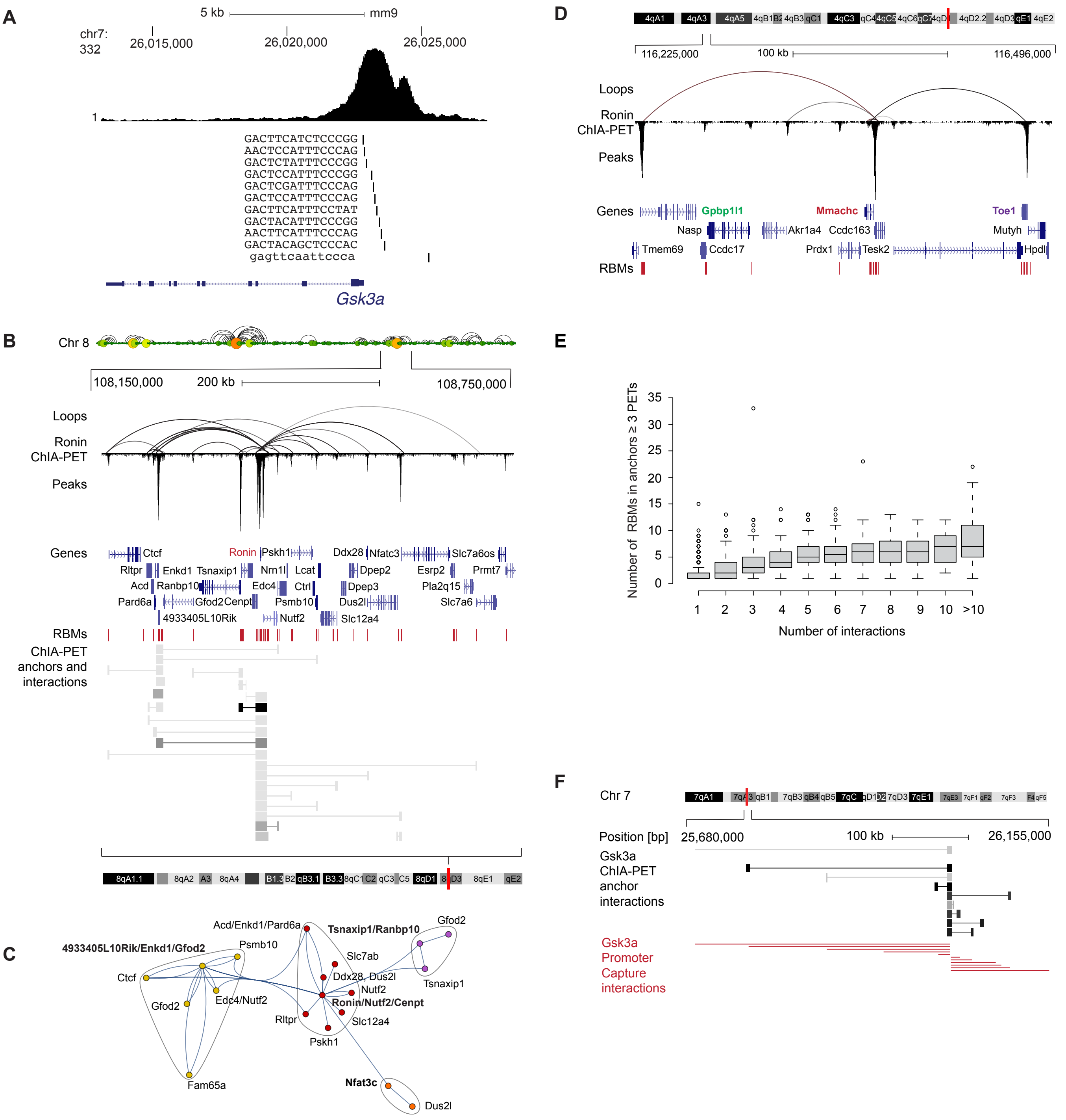

Figure S4. Ronin loops multiple genomic sites that interact *in vivo*. Related to Figure 1 and Table S1.

(A) Illustration of the Ronin-binding motifs within the Ronin binding peak at the *Gsk3a* locus.
(B) Example of a genomic region on chromosome 8 with a complex looping pattern including the Ronin locus itself. The merged interactions from two ChIA-PET experiments are shown. Interactions with more than 10 PETs are displayed that start and end in this region.
(C) Network illustration of the locus shown in (A).
(D) Example of a genomic region on chromosome 4 with a complex looping pattern involving the *Mmachc* promoter.

(E) Box plot showing the number of RBMs within individual anchors (Ronin-looped sites) versus the number of interactions. n=1388, 437, 196, 119, 78, 50, 52, 37, 19, 19, 43 from left to right.
(F) Multi-promoter assembly at the *Gsk3a* locus identified in through Ronin ChIAP-PET when compared to published promoter-capture data 69.

bp, base pairs; ChIA-PET, chromatin interaction analysis by paired-end tag sequencing; Chr, chromosome; kb, kilobase pairs; P, promoter; PET, paired-end tag.

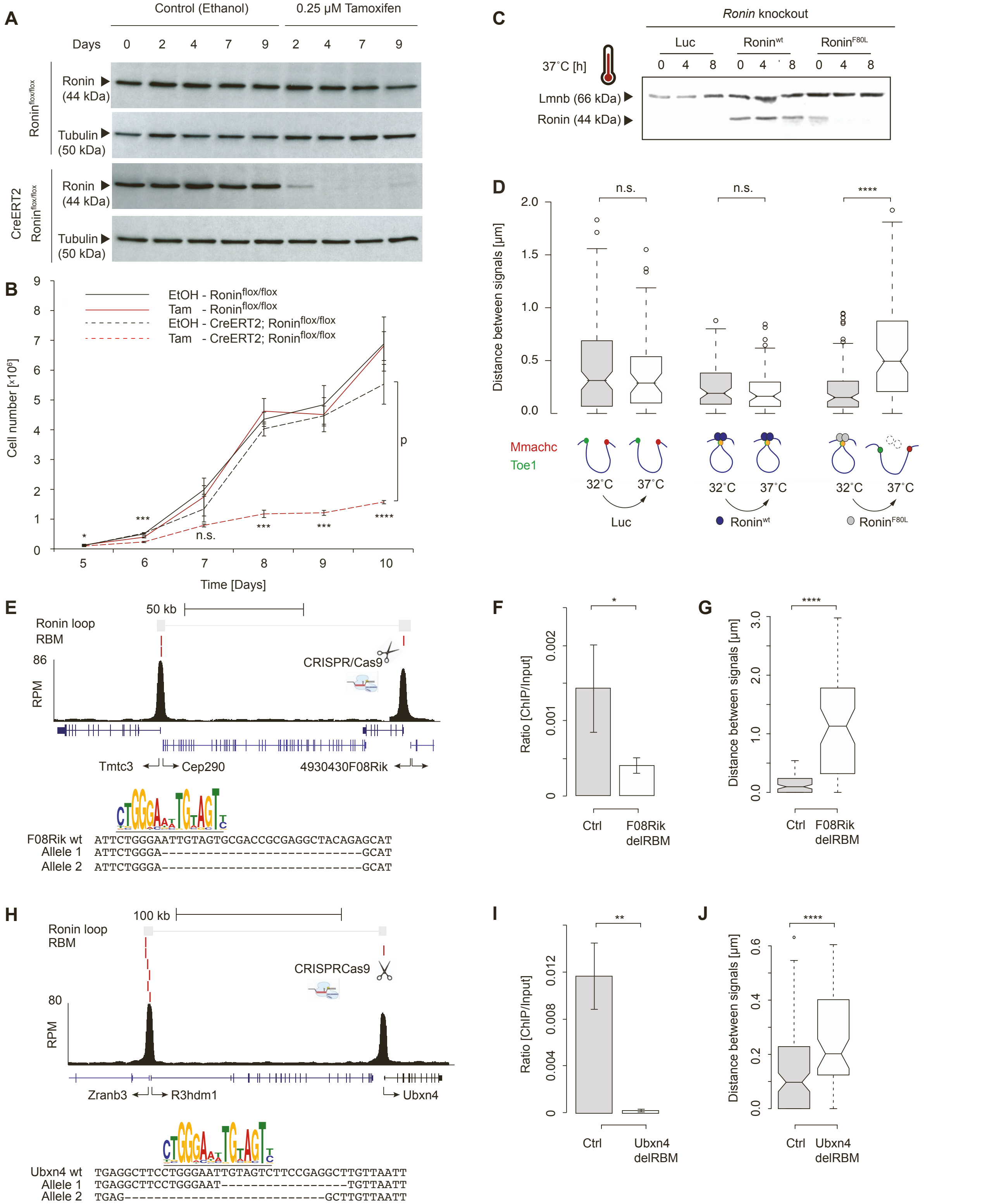

Figure S5. ***Ronin*** **knockout strategy and Ronin-looping phenotypes in cells expressing a temperature-sensitive Ronin mutant or with a deletion of the Ronin-binding motif. Related to Figure 3 and Table S1.**

(A) Western blot analysis of Ronin expression in Ronin$^{flox/flox}$ or CreERT2; Ronin$^{flox/flox}$ cells in the absence (ethanol alone) or presence of 0.25 μM tamoxifen at indicated time points. Tubulin expression was detected as loading control.

(B) Growth curves of *Ronin* knockout and control cells in the presence of 0.25 μM tamoxifen (Tam) or ethanol (EtOH). Data represent the mean ± SD. n=3; p=0.03916, 0.00059, 0.06025, 0.00017, 0.00058 from left to right. $p=2.3^{6}$ was calculated for the difference between ethanol- and tamoxifen-treated CreERT2; $Ronin^{flox/flox}$ cells.

(C) Western blot of protein lysates from *Ronin* knockout cells expressing Luciferase (Luc), Ronin wildtype ($Ronin^{wt}$) or a temperature-sensitive Ronin mutant ($Ronin^{F80L}$) after incubation at 37˚C for the indicated times. Lamin B1 (Lmnb) was detected as loading control.

(D) Box plots showing the distance between the closest green and red signals per cell measured after RNA FISH detecting the *Mmachc* and *Toe1* loci in cell types as indicated grown at 32˚C followed by 4 hours at 37˚C (bottom). n=128, 56, 88, 66, 100, 99; p=0.31564, 0.20593, $2.1x10^{11}$ from left to right.

(E) Illustration of the *4930430F08Rik (F08Rik)/Tmtc3* interaction and the alleles of the *F08Rik* locus with the RBM that was targeted by CRISPR/Cas9 in comparison to the wildtype (wt) allele.

(F) Ronin chromatin immunoprecipitation results of the *F08Rik* locus in wildtype cells and cells after CRISPR/Cas9 targeted deletion of the RBM in the *F08Rik* locus. Data represent the mean ± SD. n=4; p=0.03984 by t-test.

(G) Box plots showing the distance between the closest green and red signals per cell measured after RNA FISH detecting the *F08Rik* and *Tmtc3* loci in wildtype control cells (n=97) or cells with RBM deletion (delRBM)(n=59). Data represent the mean ± SD; $p=1.52x10^{7}$ by t-test.

(H) Illustration of the *R3hdm1/Ubxn4* interaction and the alleles of the *Ubxn4* locus with the RBM that was targeted by CRISPR/Cas9 in comparison to the wildtype (wt) allele.

(I) Ronin chromatin immunoprecipitation results at the *Ubxn4* locus in wildtype cells and cells after CRISPR/Cas9 targeted deletion of the RBM in *Ubxn4*. Data represent the mean ± SD. n=4; p=0.00217 by t-test.

(J) Box plots showing the distance between the closest green and red signals per cell measured after RNA FISH detecting the *R3hdm1* and *Ubxn4* loci in wildtype control cells (n=111) or cells with RBM deletion (delRBM)(n=66). $p=1.5x10^{7}$ by t-test.

Ctrl, control; del, deletion; F08Rik, 930430F08Rik, kb, kilobase pairs; kDa, kilodalton; RBM, Ronin-binding motif; wt, wildtype.

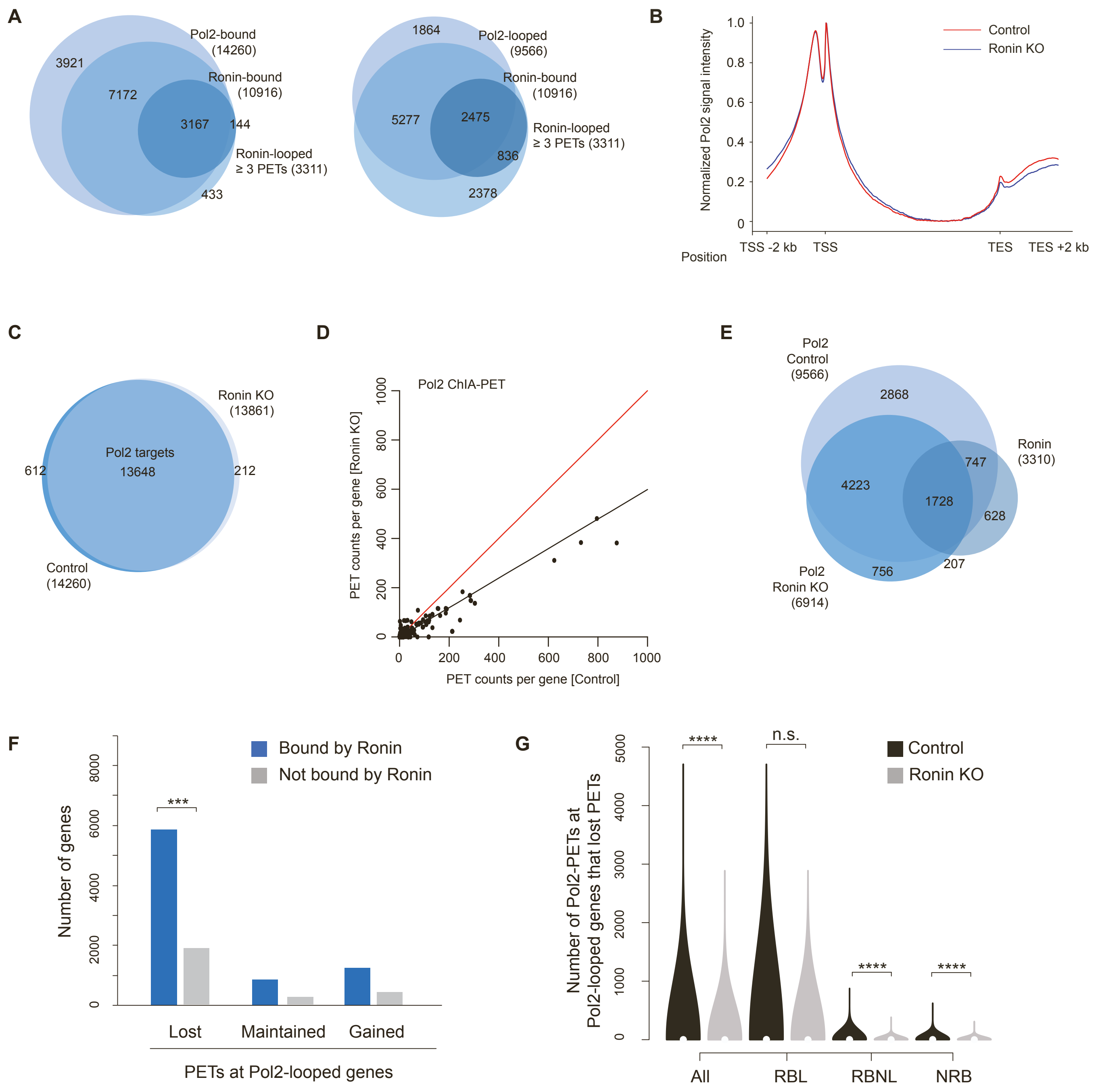

Figure S6. Ronin-mediated interactions overlap with Pol2-associated interactions that are affected after *Ronin* knockout. Related to Figure 4 and Tables S1-S3.

(A) Overlap of Pol2 targets (left) or Pol2 anchor genes (right) with Ronin targets and Ronin anchor genes.
(B) Normalized Pol2 signal intensity from 2 kb upstream of transcription start sites (TTS) to 2 kb downstream of transcription end sites (TES) of all genes in control and *Ronin* knockout ES cells.
(C) Overlap of Pol2 target genes in control and *Ronin* knockout cells.
(D) Dot plot of Pol2 ChIA-PET paired-end sequence tag (PET) counts per gene in control (x-axis) and *Ronin* knockout cells after 4 days of tamoxifen treatment (KO, y-axis). Black line corresponds to the trend line of ChIA-PET data and dashed red line represents the expected trend line if removing Ronin had no effect on chromatin interactions.

(E) Overlap of genes that are bound and looped by Ronin (Ronin anchors) in wildtype cells with Pol2-bound genes that are associated with DNA loops (Pol2 anchors) in control and or *Ronin* knockout cells.
(F) Number of genes involved in Pol2-associated DNA interactions and are bound or not bound by Ronin in wildtype cells that lost, maintained, or gained Pol2-PETs in *Ronin* knockout cells when compared with control cells.
(G) Violin plot comparing Pol2-PET counts at genes that lost Pol2-associated interactions in *Ronin* knockout cells when compared to control (Ctrl) cells that are bound and looped by Ronin (RBL, anchors), bound and not looped by Ronin (RBNL) and not bound by Ronin (NRB) in wildtype cells when compared to control cells. n=7732, 1874, 4129 and 1729 from left right; p=5.2^{7}, 0.12301, 1.2^{26} and 3.4^{11} by t-test from left to right.

Chr, chromosome; ChIA-PET, chromatin interaction analysis by paired-end tag sequencing; kb, kilobase pair; KO, Ronin knockout; Pol2, RNA polymerase II. RBM, Ronin binding motif; TSS, transcription start site; TES, transcription end site.

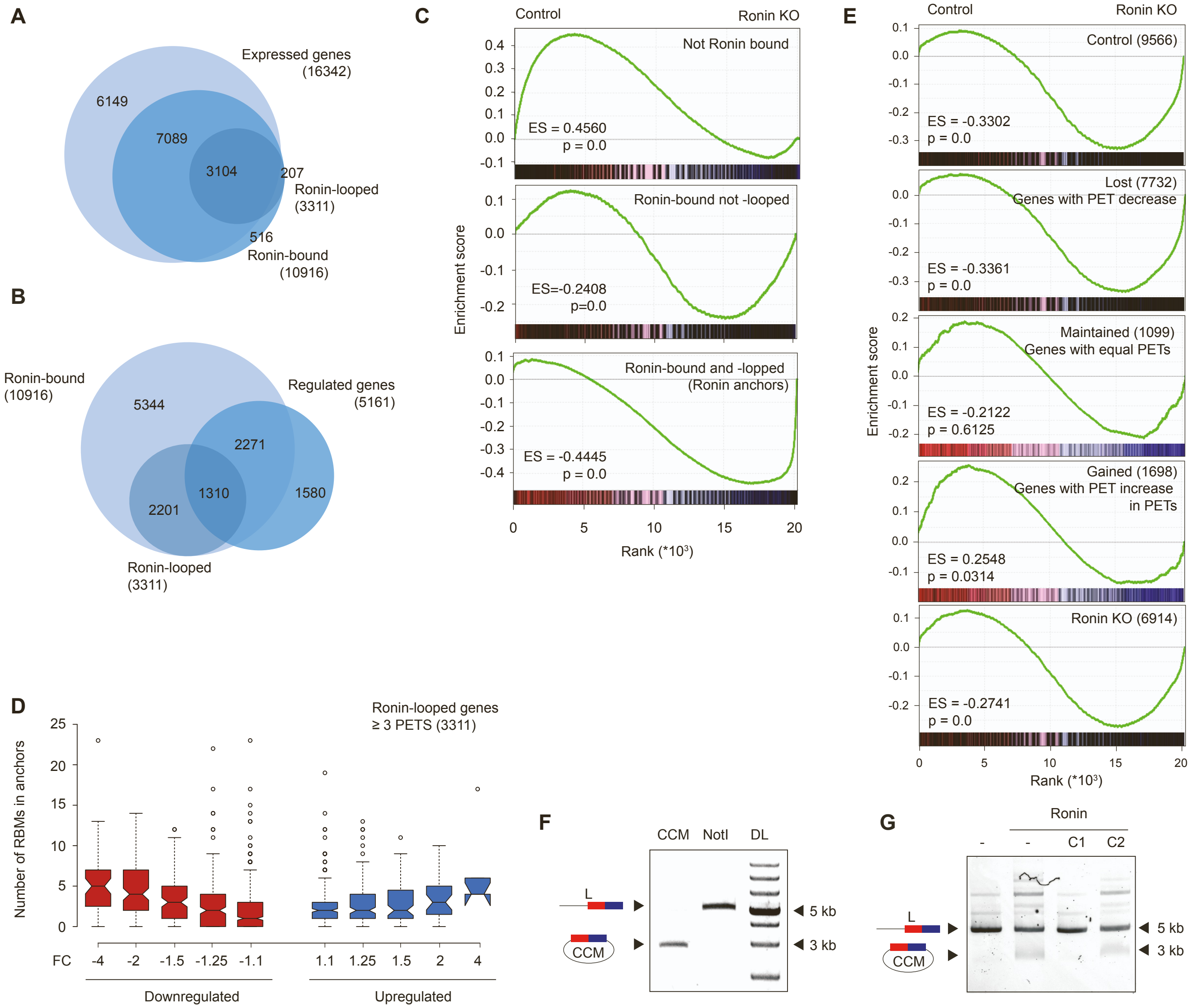

Figure S7. *Ronin* knockout-induced gene expression changes, their relation to Ronin-binding motifs and Pol2-associated looping, and circularization assay controls. Related to Figure 5 and Tables S1-S3.

(A) Venn diagram showing the overlap of Ronin-bound genes (targets) and Ronin-bound genes that are looped by Ronin (anchors, ≥ 3 PETs) with genes that are expressed (with more than 3 reads) in ES cells.
(B) Venn diagram illustrating the overlap of genes with significant changes after *Ronin* knockout (log_2-fold change after shrinkage (shlog$_2$FC) over 0.21 (FC=1.1) with Ronin target (Ronin-bound) genes and genes in Ronin anchors (Ronin-bound and Ronin-looped).
(C) Gene set enrichment analyses of genes that are not bound by Ronin (top), bound by Ronin but not looped by Ronin (0-2 PETs, middle) or genes in Ronin anchors that are bound and looped by Ronin in wildtype cells (≥ 3 PETs, bottom) after *Ronin* knockout compared with control cells.
(D) Box plot illustrating the correlation of up- and downregulated Ronin anchor genes within the indicated fold change cutoffs (sorted by shrunken log_2-fold change equivalent to the indicated fold-changes (FC) and the number of RBMs within these anchors. Each category contains the genes with a fold change equal or larger to the indicated cutoff up to the next cutoff, e.g., FC1.1=shlog$_2$FC(0.21-0.31),FC1.25=shlog$_2$FC(0.32-0.57), FC1.5=shlog$_2$FC (0.58-0.9), FC2= shlog$_2$FC(1-1.9) and FC4=shlog$_2$FC2). n=56, 117, 129, 237, 480, 217, 158, 79, 35, 5 from left to right.

(E) Gene set enrichment analyses of genes that are associated with Pol2 anchors in control (top) and Ronin knockout cells (bottom) and those that lost, maintained, or gained Pol2-associated interactions (Pol2-PETs) after *Ronin* knockout (middle).

(F) NotI-restriction digest to confirm the “3 kb” circular covalently closed monomer (CCM) after Ronin-catalyzed ligation of the RBM-containing linear DNA substrate (pTZ1860) used in the circularization assays shown in Fig.1.

(G) Circularization assay in the absence and presence of competitor DNA containing the Ronin-binding motif (C1), or random nucleotides (C2).

DL, DNA Ladder; ES, enrichment score; FC, fold change; kb, kilobase pairs; KO, knockout; L, linear; NRB, Not Ronin bound; PET, paired-end tag.
